# A Van Gogh/Vangl tyrosine phosphorylation switch regulates its interaction with core Planar Cell Polarity factors Prickle and Dishevelled

**DOI:** 10.1371/journal.pgen.1010849

**Published:** 2023-07-18

**Authors:** Ashley C. Humphries, Claudia Molina-Pelayo, Parijat Sil, C. Clayton Hazelett, Danelle Devenport, Marek Mlodzik

**Affiliations:** 1 Dept. of Cell, Developmental, & Regenerative Biology,Graduate School of Biomedical Sciences, Icahn School of Medicine at Mount Sinai, New York, New York, United States of America; 2 Dept. of Molecular Biology Princeton University, Princeton, New Jersey, United States of America; NYU School of Medicine, UNITED STATES

## Abstract

Epithelial tissues can be polarized along two axes: in addition to apical-basal polarity they are often also polarized within the plane of the epithelium, known as planar cell polarity (PCP). PCP depends upon the conserved Wnt/Frizzled (Fz) signaling factors, including Fz itself and Van Gogh (Vang/Vangl in mammals). Here, taking advantage of the complementary features of *Drosophila* wing and mouse skin PCP establishment, we dissect how Vang/Vangl phosphorylation on a specific conserved tyrosine residue affects its interaction with two cytoplasmic core PCP factors, Dishevelled (Dsh/Dvl1-3 in mammals) and Prickle (Pk/Pk1-3). We demonstrate that Pk and Dsh/Dvl bind to Vang/Vangl in an overlapping region centered around this tyrosine. Strikingly, Vang/Vangl phosphorylation promotes its binding to Prickle, a key effector of the Vang/Vangl complex, and inhibits its interaction with Dishevelled. Thus phosphorylation of this tyrosine appears to promote the formation of the mature Vang/Vangl-Pk complex during PCP establishment and conversely it inhibits the Vang interaction with the antagonistic effector Dishevelled. Intriguingly, the phosphorylation state of this tyrosine might thus serve as a switch between transient interactions with Dishevelled and stable formation of Vang-Pk complexes during PCP establishment.

## Author summary

Tissues and organs are generally polarized along two axes. In addition to apical-basal epithelial polarity, tissues have often polarized cells or groups fo cells within the plane of the tissue, the so-called Planar Cell Polarity or PCP for short. PCP depends upon a conserved set of so-called core factors, which are conserved across the animal kingdom. The orientation of these factors and hence the cellular polarity also depends on the secreted Wnt-ligands. The key transmembrane core PCP factors, and thus main actors in the context are Frizzled (Fz) and Van Gogh (Vang/Vangl). We have identified a specific post-tranlational modification, a tyrosine phosphorylation of Vang/Vangl, which regulates the signaling outcome by directing with which cytoplasmic PCP factor Vang/Vangl will physically associate. Hence this phosphorylation event can be called a “phosphorylation switch”, which guides the transition of intermediary complex formation of Vang and Fz complexes, to the stable, polarized complexes anchored in this case by Vang/Vangl proteins. Interestingly, the formation of the unstable, intermediary complexes is equally important to the polarization process as is the final stable complex establishment.

## Introduction

Cellular polarization is critical for the morphogenesis and function of organs and most tissues during development, with perturbation of cellular polarity and tissue organization implicated in numerous diseases. In particular, epithelial cells can be polarized in two axes: the ubiquitous epithelial polarity in the apical-basal axis, and polarity in the plane of the epithelium, referred to as planar cell polarity (PCP) (reviewed in [1–7]). Although PCP was initially discovered in epithelia, the cellular mechanisms controlling PCP are detected in many other cell types, including migratory mesenchymal cells or neurons (reviewed in [3,8,9]. In this study we focus on PCP in epithelia. PCP establishment is mainly governed by members of the conserved Wnt/Frizzled-PCP pathway, referred to as the core PCP pathway (reviewed in [1–7])

The core PCP factors were all originally discovered in *Drosophila* but are highly conserved across metazoa. PCP complexes at the cell surface include the atypical seven-pass transmembrane (TM) cadherin Flamingo (Fmi; Celsr in mammals), the seven-pass TM protein Frizzled (Fz; Fzd in vertebrates with several family members), and the four-pass trans-membrane protein Vang (Vangl1 and Vangl2 in mammals; a.k.a. *strabismus/stbm* in *Drosophila* and *Xenopus*). These TM proteins recruit the core cytoplasmic PCP factors Dishevelled (Dsh; Dvl1-3 in mammals), Diego (Dgo; Inversin/Diversin in vertebrates), and Prickle (Pk1-3) (e.g. reviewed in [1–7]). The initial stage of PCP signaling results in asymmetric localizations of its core members. These become enriched into two complexes on opposite sides of a given cell, which generate an intracellular bridge to convey polarity across cell membranes from cell to cell across the tissue (see reviews above). The resulting complexes, anchored in the membrane as Fz-Fmi on one side and Vang-Fmi on the other, stabilize each other intercellularly (between cells) and antagonize each other intracellularly (within each cell). The intracellular antagonistic behavior of these complexes is mediated by the cytoplasmic PCP factors Dsh/Dvl, Dgo, and Pk. The final asymmetric localization is generated through a dynamic process, and thought to include transient intermediary subcomplexes (reviewed in [1–3,5–10]). Asymmetric core PCP complex localization then directs spatially restricted downstream signaling events through cell-type specific effectors, leading to cytoskeletal rearrangement, centriole/centrosome/ciliary positioning, migratory regulation, and/or nuclear read-outs (see reviews above).

Asymmetric distribution of PCP complexes is a direct consequence of their interactions during polarity establishment. It is observable in several tissues, ranging from cells of the *Drosophila* wing, where these complexes align to the proximal-distal axis, to for example mouse skin, where they align in the antero-posterior axis [11–14], also reviewed in [1,3–5,15]. Molecular interactions promote the formation of stable complexes at proximal (*Drosophila* wing) or anterior (mouse skin) cell membranes (Fmi/Celsr1-Vang/Vangl-Pk) and the equivalent distal or posterior cell surfaces (Fmi/Celsr1-Fz/Fzd-Dsh/Dvl), with Fmi (Celsr1 in mammals) forming a homotypic interaction across cells, which stabilizes these complexes intercellularly. Simple epithelia like the *Drosophila* wing display not only such highly coordinated PCP complex localization logic, but also an obvious and simple PCP read-out, with the formation of a single-actin based hair at the distal vertex of each cell pointing distally. Disruption of PCP establishment in the wing is easily observable by misorientation of the cellular hairs within the field of cells, or the formation of multiple cellular hairs in a single cell (reviewed in [1,4,5,15].

Although the formation of PCP is still best studied in *Drosophila*, where the core factors were initially discovered and functionally dissected [1,4,5,15], the importance of PCP during vertebrate development and human disease has become widely recognized (reviewed in [2,3,6,7,10,16,17]). For example, PCP directs polarized ciliary beating to generate fluid flow in the embryonic node, trachea, oviduct and brain ventricles. Failure to generate coordinated fluid flow in PCP mutant mice leads to left-right patterning defects, defective mucociliary clearance, sterility and hydrocephalus [7,18–24]. Core PCP genes are essential for neural tube closure in mammals and pathological variants in PCP genes are strongly associated with neural tube defects in humans [25–27]. A notable example of PCP in mammals is the uniform alignment of body hairs across the skin surface, where the core PCP proteins direct polarized morphogenesis and tissue-wide alignment of hair follicles [14,28–30].

Among the core PCP factors the Vang/Vangl family proteins occupy a unique role, as they have been shown to physically interact with all other 5 core PCP factors (rev in [1,4,5,15]) and also the A/B-polarity protein Scribble (Scrib) [31,32], with Dsh/Dvl, Pk, Dgo, and Scrib binding to the cytoplasmic C-terminal tail of Vang/Vangl (e.g. see [12,33]). Similarly, Vang/Vangl proteins have been shown to associate with the transmembrane factors Fmi/Celsr in cis and Fz in trans [14,34–36]. *Drosophila Vang* was identified by its strong PCP loss-of-function defects in wing and eye screens, and was also demonstrated to cause a domineering non-autonomous phenotype affecting nearby wild-type cells through propagation of aberrant polarity from mutant cells outward [37,38]. Vang/Vangl genes encode four-pass transmembrane proteins with intracellular cytoplasmic amino- and carboxy- terminal regions. All its cytoplasmic interactions have thus far been physically mapped to the C-terminal tail. It requires Pk and Scrib interactions for the formation of its stable complex. While it can also interact with Dsh and Dgo, these latter interactions are thought to be more transient and antagonistic to stable complex formation, with for example Dsh/Dvl binding to Vang thought to antagonize mislocalized Vang protein and/or prevent a Vang-Pk interaction in a wrong cellular location [12,13,31–33]. Its mammalian homologs Vangl1-2 regulate all PCP processes studied in higher organisms (for example [32,39,40] reviewed in [41,42]). Mutations of both *Vangl1* and *Vangl2* have also been identified in human patients affected with *spina bifida* and *craniorachischisis* [43] and subsequently shown to affect PCP signaling and establishment, using the well established *Drosophila* wing PCP model [44].

It was previously suggested that phosphorylation of Vang/Vangl proteins might be an important regulatory mechanism of core PCP complex formation. Along these lines, it was shown that Vang/Vangl proteins are phosphorylated on serine/threonine residues in the N-terminal tail, which is thought to affect complex formation and stability [45–47]. In order to better define the mechanisms underlying Vang/Vangl regulation and its interactions with downstream PCP factors, we investigated how phosphorylation of Vang/Vangl might regulate its function. Here we describe a concerted effort using the *Drosophila* wing and mouse skin models to better define potential Vang/Vangl interactions regulated by tyrosine phosphorylation. We have identified specific tyrosine phosphorylation events via mass-spectroscopy analyses. Here, we focus on a conserved phosphorylated C-tail tyrosine, which resides within the broad region of Pk and Dsh/Dvl binding. We fine-mapped Pk and Dsh binding to identify the amino acids required and find that Pk and Dsh binding sites overlap. Strikingly, the defined phosphorylation site resides in this newly defined overlapping binding regions of Pk and Dsh, and it regulates Vang/Vangl interactions with these core factors. Pk binds preferentially to the phosphorylated state, and Dsh to the unphosphorylated region. Our functional *in vivo* rescue studies demonstrate that binding of Vang to both effectors is physiologically relevant, as all single point mutations, which allow selective binding to either Pk or Dsh fail to rescue the *Vang* null mutant phenotype, as evident both in pupal wings and in adult tissue. Not surprisingly based on previous reports [12,33,48], membrane association of Pk, albeit reduced, is not lost in the Pk binding Vang mutants, confirming a more complex Pk recruitment scenario. With our defined single point mutation for Dsh binding, we show the physiological relevance of the Dsh interaction with Vang for the first time. Overall, we have identified new regulatory means for binding to antagonistic effectors during PCP establishment via a phosphorylation switch at a conserved site.

## Results

### Vang/Vangl2 proteins display a multitude of phosphorylations *in vivo*

Several studies have demonstrated that *Drosophila* Vang and mouse Vangl2 are phosphorylated on serine (S) and threonine (T) residues, and a functionally important S/T phosphorylation cluster has been defined within the N-terminus [45–47,49]. In addition, phosphorylation on a specific tyrosine (Y) has been shown to be important for correct Vangl2 trafficking in mammalian cells and *Drosophila* Vang *in vivo*, respectively [47,50].

**Fig 1:**
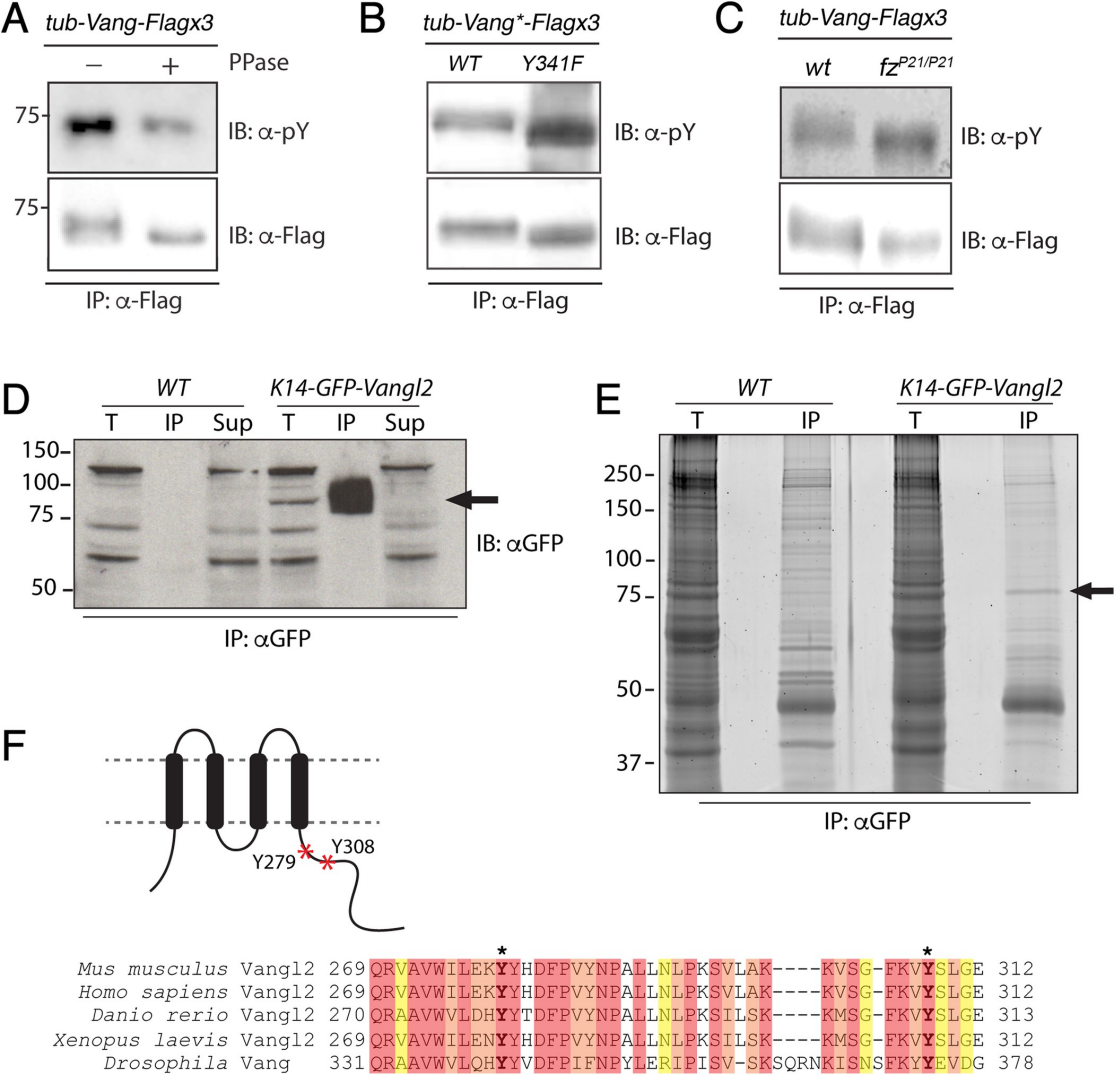
Vang is tyrosine phosphorylated in vivo. (**A-C**) Western blots of lysates from pupal wing discs showing that Vang is tyrosine phosphorylated *in vivo*. Note the reduction in signal of anti-pY with PPase treatment (**A,** upper panel) and the corresponding loss of a band shift (lower panel). (**B**) Tyrosine phosphorylation is maintained in the Vang-Y341F protein, suggesting other tyrosines are phosphorylated; and tyrosine phosphorylation is independent of Fz, as anti-pY staining is not affected in homozygous mutants *fz*^*P21*^ null animals (**C**). **(D)** Immunoprecipitation of GFP-Vangl2 from E15.5 wild-type control (*WT*) and K14-GFP-Vangl2 mouse epidermal lysates. Western blot using anti-GFP antibodies detects an ~85KD band corresponding to the GFP-Vangl2 fusion protein (arrow). T = total protein input, IP = immunoprecipitate, Sup = supernatant. **(E)** Anti-GFP immunoprecipitates from wild-type control (*WT*) and K14-GFP-Vangl2 mouse epidermal lysates run on an SDS-PAGE gel stained with SPYRO-Ruby to detect total protein. The ~85KD band present in the K14-GFP-Vangl2 but not wild type control immunoprecipitate was excised and processed for mass spectrometry. (**F)** Schemtic cartoon of mouse Vangl2 and sequence alignment of mVangl2 with human, zebrafish, *Xenopus*, and *Drosophila* orthologs. The region spanning amino acids 269–312 in mVangl2 within the C-terminal cytoplasmic tail is shown. Mass spectrometry analysis detected phosphorylation at highly conserved tyrosine residues 279 and 308 of mouse Vangl2 (bold with asterisk). The position of these is also marked with red asterisks in the cartoon.

Our initial analyses performed in the Kelly et al. (2016) study [45–47,49] strongly suggested that additional Y residues are also likely to be phosphorylated. To better define the role of tyrosine phosphorylation in Vang/Vangl function, we first verified that *Drosophila* Vang is tyrosine phosphorylated *in vivo* (Fig 1A). Immunoprecipitation of Vang-Flagx3 from larval wing discs, showed a positive phospho-tyrosine signal, which was reduced upon phosphatase treatment (Fig 1A; note that a down-shift in the mobility of Vang consistent with successful phosphatase treatment was observed). Second, Vang remained Y-phosphorylated in the Vang-Y341F mutant (Fig 1B). As this site has been shown to be phosphorylated, with Y341 (Y279/280 in mouse *Vangl2*) promoting correct membrane trafficking [47,50], these data suggested that other tyrosines must be phosphorylated in Vang proteins *in vivo*.

We furthermore detected Y-phosphorylation of Vang in a *fz*- null mutant background (Fig 1C), indicating that at least some Vang Y-phosphorylation is Fz independent, unlike the N-terminal Vang/Vangl2 S/T-cluster phosphorylation. S/T cluster phosphorylation is Fz dependent and causes a detectable band shift on protein gels (see Fig 1C for loss of band shift; see also [46,47,49]). Taken together, these *in vivo Drosophila* data suggest that Vang is phosphorylated on tyrosine residue(s) outside the defined Y341 and that at least some of these additional phosphorylation events might be Fz independent.

**Fig 2:**
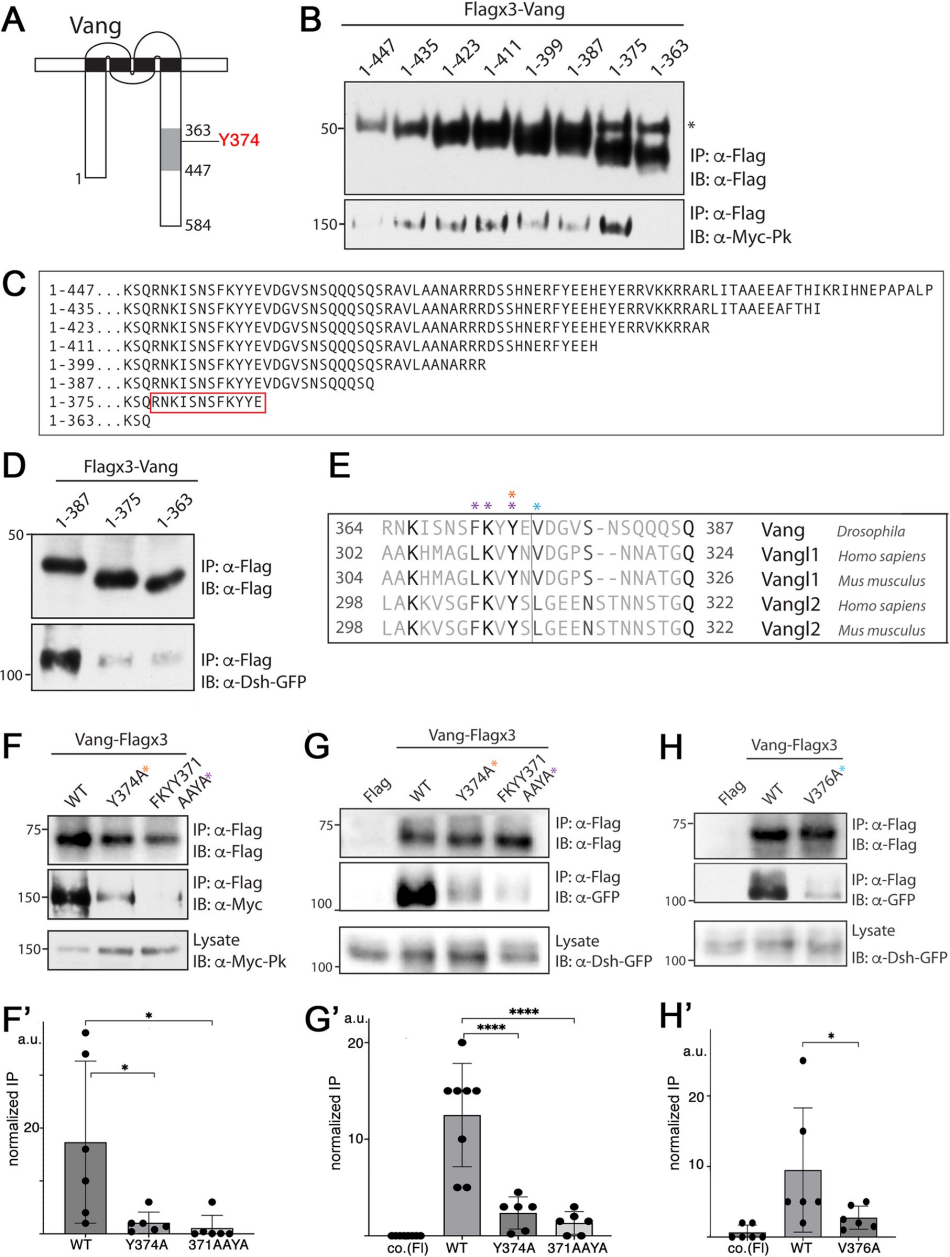
Pk and Dsh bind to adjacent, partially overlapping regions in the C-terminal tail of Vang. **(A)** Schematic of Vang showing the previously mapped binding region of Pk and Dsh within its C-terminal tail (shaded in gray), residues 363–447 [53]. Y374 is indicated in red (this residue is equivalent to Y308 in mouse Vangl2, see alignment in Fig 1F). Note that Y374 is located within the shaded region. (**B**) Western blot showing binding between Myc-Pk and C-terminal truncations of Flagx3-Vang, using a series of C-terminal truncations within residues 363–447. Binding is retained up until the 1–363 truncation, defining residues 363–375 in Vang as critical for its binding to Pk. (**C**) Schematic of sequences of the Vang C-terminal truncations as used in (B) and (D), red box highlights amino acids required for Pk-binding. (**D**) Western blot showing binding between Dsh-GFP and C-terminal truncations of Flagx3-Vang. Note that binding is retained up until the 1–387 truncation and markedly reduced in the 1–375 truncation and shorter. (**E**) Sequence alignment showing the conservation of amino acids in the *Drosophila* Vang 364–387 region with mouse and human Vangl proteins. Colored asterisks highlight amino acids mutated in binding experiments shown in panels F, G and H. (**F**) Western blot showing binding between Myc-Pk and selected Vang-Flagx3 mutants as indicated. Colored asterisks refer to specific amino acids in sequence schematic in (**E**) mutated in the experiment. Note marked reduction in binding for the single mutant (Y374A) and almost complete loss of binding in the triple mutant FKYY371AAYA. (**F’**) Quantification, derived from 6 independent replicates, and statitistical analysis of binding differences. * *p*<0.05 as determined with Anova (and Tukey’s post test to compare all samples with each other). (**G**) Western blot showing binding between Dsh-GFP and the indicated Vang-Flagx3 mutants. Note the reduction in binding follows a similar pattern to what was observed with Pk (compare to panel **F**), the equivalent Y residue to Y374 is detected as phosphorylated in mouse Vangl2 (see Fig 1D–1G). (**G’**) Quantification, combined from 6–7 replicates, and statitistical analysis of binding differences. **** *p* <0.0001 as determined with Anova (and Tukey’s post test to compare all samples). (**H**) Western blot showing binding between Dsh-GFP and Vang-Flagx3 with the V376A mutant. Note a marked reduction in binding. V376 is at the junction to the Pk-binding region (blue asterisk in **E**), suggesting an overlap in binding regions between Pk and Dsh. It was the only single residue mutation in the 376–387 stretch to affect Dsh binding. (**H’**) Quantification, combined from 6 replicates, and statitistical analysis of binding differences. * *p* <0.05 as determined with Anova.

To gain insight into which tyrosines might be phosphorylated, we turned to a mass spectrometry-based approach. As it is technically challenging to obtain sufficient material from *Drosophila* pupal wings for such mass spectrometry studies [51], we used mouse Vangl2 from embryonic skin. Due to its abundance and accessibility, the skin epidermis is an excellent model for biochemical analyses of proteins in their native context. To identify post-translational modifications on epidermally expressed Vangl2, we performed an IP-MS analysis of GFP-Vangl2 protein purified from the skin of *K14-GFP-Vangl2* transgenic embryos [52]. Protein lysates were prepared from skin samples dissected from embryos at E15.5, and GFP-Vangl2 was immunoprecipitated using GFP-antibodies (Fig 1D). A prominent band of ~85KD was present in immunoprecipitates from *K14-GFP-Vangl2* embryos but not wild type controls. Gel fragments containing protein in the 85-90KD range were excised and processed for LC-MS/MS analysis (Fig 1E). Over 220 unique, high confidence peptides were recovered spanning >86% of the GFP-Vangl2 fusion protein. Importantly, phosphorylation modifications were detected at two different tyrosine residues Y279 and Y308 (Fig 1F), both of which are conserved across vertebrates and in *Drosophila* (Figs 1F, and S1 and S1 Table). Y279 is located within Vangl2’s TGN-sorting motif and is equivalent to Y341 in *Drosophila*, whose phosphorylation was previously implicated in Vangl2 trafficking [47,50], indicating that our methods were able to detect functionally relevant Vang/Vangl2 phospho-tyrosine modifications. Intriguingly, Y308 corresponds to residue Y374 in *Drosophila*, which is located within the previously defined Dsh and Pk binding region of Vang [53] (Figs 1F and 2A). The strong conservation of this tyrosine and its position within the Dsh/Dvl and Pk binding region (see below) suggested that modification at this site may be important for Vang/Vangl function.

### Vang binds Pk and Dsh at an overlapping region around the Y374/Y308 phospho-site

Vang/Vangl proteins have been shown to physically interact *in vitro* with all other members of the core PCP factor group. The molecular interactions of Vang with the cytoplasmic core factors have previously been mapped to specific regions within the C-terminal tail of the protein (shaded C-tail stretch in Fig 2A): amino acids 363–447 are required for its interaction with Pk and Dsh [53], a region which contains a phosphorylated conserved tyrosine as identified in our mass spec study (Fig 1D–1F, schematic in Fig 2D). We thus aimed to refine the binding sites of Pk and Dsh within this region and define the potential role of the phosphorylated Y-residue in this context.

We first made a series of C-terminal truncations of *Drosophila* Vang covering the known binding region [53], amino acids 363–447 in the C-terminal tail, and reduced this binding domain by a series of 12 amino acid truncations (Fig 2B and 2C). Pull-down experiments, performed in S2 cells, revealed that Pk could interact with all such C-terminal Vang truncations with the exception of 1–363, which suggested that amino acids 364–375 were required for Pk-binding (Fig 2B, and see 2C for sequence schematic of deletion constructs). Importantly, this amino acid stretch contains the Y residue, identified as phosphorylated *in vivo* in the mass spec studies (see above), and conserved surrounding residues. We next assessed Vang interactions with the other cytoplasmic effectors that were also previously shown to interact within the C-tail region [33,53]. Strikingly, Dsh also showed markedly reduced binding when residues 363–387 were deleted (Fig 2C and 2D). In the case of Dsh we observed binding loss with the 1–375 truncation, directly adjacent and overlapping to the Pk binding area, suggesting that some amino acids within 376–387 stretch are critical for the Vang-Dsh interaction (Fig 2C and 2D). Taken together, these data suggest that Pk and Dsh bind to regions immediately adjacent to each other, and likely overlapping.

This region of Vang/Vangl2 contains the conserved phosphorylated tyrosine, Y374 in *Drosophila* and Y308 in mouse Vangl2 (Fig 2E), we thus tested whether this Y-residue and/or associated conserved residues affect interaction with either Pk or Dsh or both. For example, we wished to ask whether binding could be charge or aminoacid structure (aromatic ring) dependent. A Vang-Y374A substitution (which removes both structure and potential charge) caused a markedly diminished binding of both Pk and Dsh (Fig 2F and 2G, respectively, see quantification in 2F’ and 2G’). This effect was further enhanced in the triple mutant affecting the whole FKxY motif (FKYY371AAYA) with interaction of either Pk or Dsh further reduced (Fig 2F, 2F’, 2G and 2G’). It is worth noting that in control experiments with the core PCP effector Dgo or the apical-basal polarity protein Scribble (Scrib), which have both been shown to interact with the C-tail of Vang/Vangl2 [13,31–33], mutations in the conserved FKYY residue stretch of Vang did not affect their binding to Vang (S2 Fig). Furthermore, the specificity and importance of Y374 and the FKYY motif for the interaction with Pk and Dsh within the defined binding region was confirmed via additional point mutations in the same region. For example, mutations of K366, F371, or K372 did not demonstrate a detectable requirement for Pk binding (S2 Fig).

Taken together, these data suggest that Pk and Dsh, two antagonistic cytoplasmic core PCP factors, share an overlapping binding site centered around a conserved phosphorylated tyrosine (Y374 in *Drosophila* and Y308 in mouse Vangl2) in the C-terminal tail region of Vang proteins with both Pk and Dsh requiring Y374 for binding within a conserved stretch of amino acids.

To further refine the binding requirement(s) of the Vang-Dsh interaction within the neighboring residues 376–387 (necessary in the truncation series for Dsh binding to Vang, Fig 2D), we scanned the respective amino acids with single alanine substitutions. This revealed surprisingly that only one substitution to alanine (A), that of a partially conserved valine (V) at position 376 (see alignment in Fig 2E), V376A, led to a marked reduction in Dsh binding (Fig 2H and 2H’). Notably, this residue is directly adjacent to the region required for Pk binding, but we did not observe an impact of V376A on the Vang-Pk interaction (S2 Fig). Thus, in addition to the above conclusion regarding the Y374 motif, our data also suggested that we have defined a specific point mutant at V376, which is only partially conserved (Fig 2E), that specifically modulates Vang-Dsh binding (see also below).

### Y374 phosphorylation/charged state contributes to effector binding regulation

As the key residue required for binding to both Pk and Dsh was a phosphorylated tyrosine, we thus asked whether phosphorylation—or charge associated with phosphorylation—at this site might regulate binding. We first performed substitution of Y374 to phenylalanine (F), with Y374F retaining the structural aromatic ring associated with tyrosine, but it remains uncharged as it cannot be phosphorylated. Conversely, Y374D or Y374E substitutions maintain charge, but they change the structure of the residue (loss of aromatic ring). Importantly, these substitutions also allowed comparison to the Y374A scenario, which diminished binding of both Pk and Dsh (Fig 2F–2G’).

**Fig 3:**
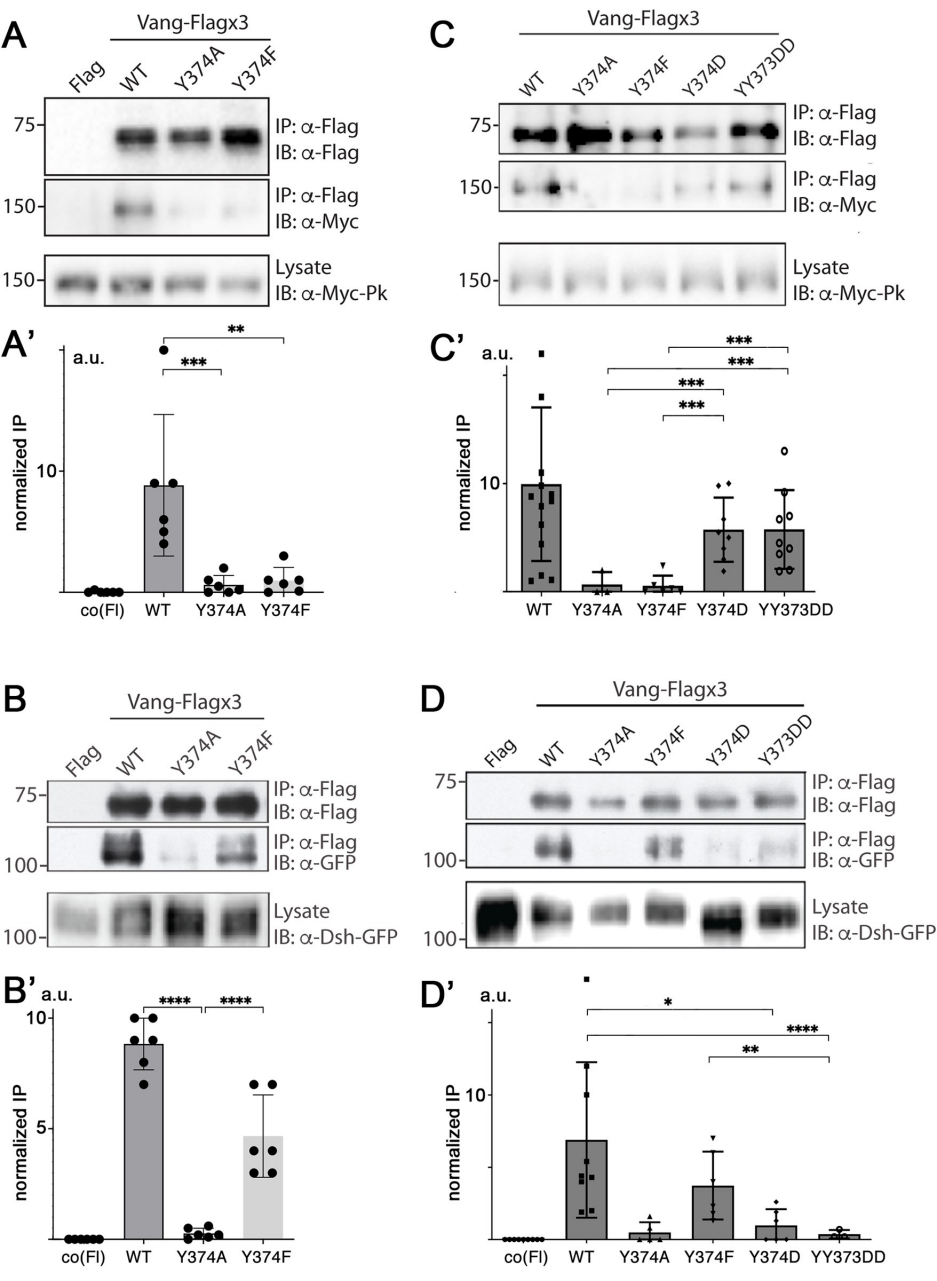
Differential substitutions at Y374 reveal a role for charge/phosphorylation in binding regulation. (**A**) Western blot showing binding between Myc-Pk and the indicated Vang-Flagx3 mutants, Y374A and Y374F. Note binding is markedly reduced in both cases. (**A’**) Quantification, combined from 6 independent replicates, and statitistical analysis of binding differences. **p <0.005, and ***p<0.0005 as determined with Anova (and Tukey’s post test to compare all samples with each other). (**B**) Western blot showing binding between Dsh-GFP and the indicated Vang-Flagx3 mutants. Note binding is reduced with an alanine substitution, Y374A, but not with phenylalanine, Y374F, which retains the aromatic ring feature of tyrosine. (**B’**) Quantification, combined from 6 independent replicates, and statitistical analysis of binding differences: ****p<0.0001, determined with Anova (and Tukey’s post test). (**C**) Charged amino acid substitutions of Y374 partially restore Pk binding. Western blot showing binding between Myc-Pk and the indicated Vang-Flagx3 Y374 substitutions. Note that Y374D and YY373DD recover significant binding of Pk, as compared to Y374A and Y374F. (**C’**) Quantification, combined from 4–10 replicates, and statitistical analysis of binding differences. ***p<0.0005 as determined with Anova (and Tukey’s post test). (**D**) Charged amino acids interfere with the Dsh binding to Vang. Western blot showing binding between Dsh-GFP and the indicated Vang-Flagx3 substitutions. Note that Vang Y374F is by far the best interacting mutant, again confirming a requirement of an aromatic ring feature/structure for Dsh binding (see also panel **B**). (**D’**) Quantification, combined from 3–9 independent replicates, and statitistical analysis of binding differences. *p<0.05, **p <0.005, and ****p<0.0001 as determined with Anova (and Tukey’s post test).

In these binding studies, Pk showed equally diminished binding to both Y374A and Y374F (Fig 3A, quantified in 3A’), while Dsh retained binding to Y374F (Fig 3B and 3B’; note Dsh-binding was reduced in the Y374A substitution in the same experiment as a control, cf. to Fig 2G and 2G’). These data suggested that Dsh binding requires the structure of the amino acid, an aromatic ring, shared between tyrosine and phenylalanine, but is not impacted by phosphorylation or charge of this site. To ask more directly whether a charge at Y374 can contribute to binding regulation, we tested a Y374D, Y373DD, and Y374E substitutions (with D and E, being charged residues, to mimic a partial phosphorylation charge). While Pk displayed significant binding to Y374D and Y373DD (Fig 3C, quantified in Fig 3C’, compare to Y374F and Y374A controls in same panels) as well as to Y374E (S3A Fig), Dsh bound poorly to these charged substitution mutations (Fig 3D and 3D’, compare to wt-control and Y374F in same panels; and S3B Fig). Taken together, these data suggested that Dsh requires an aromatic ring at the binding region centered on Y374 and its binding might be inhibited by charge, while the Vang-Pk interaction appeared to be dependent on the negative charge, consistent with the notion that it would be promoted by phosphorylation.

**Fig 4:**
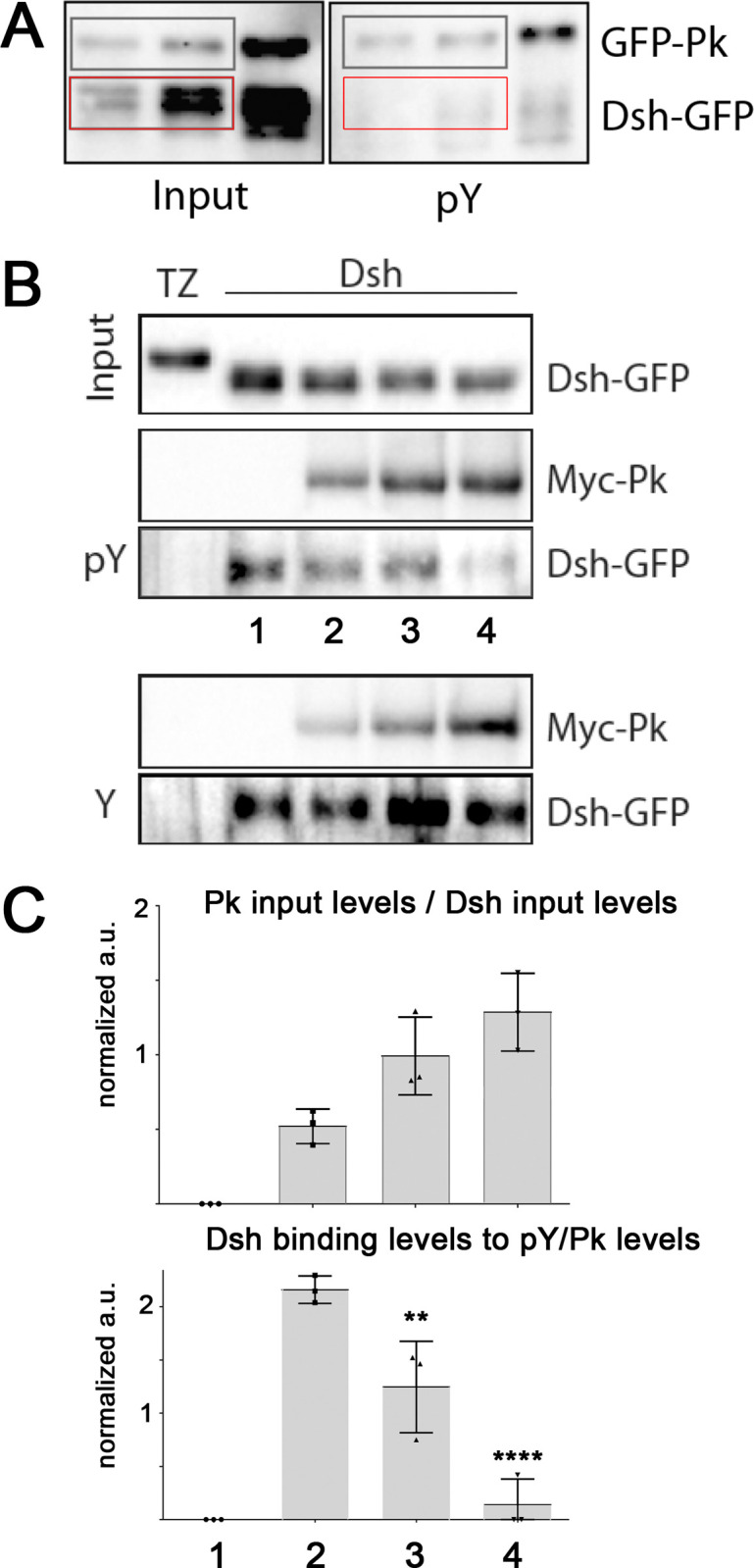
A phospho-peptide of Y374 surronding sequences binds preferentially to Pk. (**A**) Pk interacts better with/binds to the phosphorylated “pY” peptide (S4 Fig for peptide sequence). Compare bands between input and pY-bound in greay boxes for Pk vs. for Dsh (red boxes). (**B**) Binding of Dsh (Dsh-GFP) is outcompeted by Pk (myc-Pk) on the “p-Y” (phospho-peptide), but not the non-phosphorylated (“Y”) peptide (bottom panel). Western blot showing retention of Dsh-GFP on beads coupled with the respective peptide, either “pY” for “Y” among mixing with Pk. Upper blot shows stable Dsh-GFP input, TZ is an unrelated control protein. Note reduced binding of Dsh-GFP when mixed in solution with increasing levels of Pk in the context of pY peptide conjugated beads (middle panel), but not the Y peptide (bottom panel). (**C**) Quantification of binding competition as shown in (**B**), combined from 3 independent replicates. Increasing Pk levels in the solution (upper graph) caused a decrease in Dsh binding to the “pY” peptide (lower graph). Statitistical analysis of binding differences (to lane #2): **p <0.005, and ****p<0.0001 (determined with one-way Anova).

To confirm this hypothesis, we also generated the equivalent binding region as an *in vitro* peptide and phospho-peptide. The peptide generated encompasses the Vang region 367–381 that the cell based experiments predicted to be sufficient to bind to both Pk and Dsh, in its phosphorylated form at Y374, ISNSFKY[pY]EVDGVSN-amide (pY-peptide) and as a non-phosphorylated control (Y-peptide, see S4A Fig for peptide sequence). These peptide interaction assays could test whether the binding region defined above (Fig 2) is sufficient for effector binding, not just necessary, and whether phosphorylation influences the Pk interaction. The caveat of these purely *in vitro* association assays is however that these are rather artificial and mixing peptides with purified proteins in a test tube certainly misses the cellular physiological regulation. Nevertheles, and consistent with the co-IP studies, the (phosphorylated) pY-peptide preferentially interacted with Pk (Fig 4A). To further corroborate this, we assayed peptide interactions with Pk and Dsh in a competitive binding assay, revealing that Dsh-GFP protein when mixed with the pY peptide (coupled to beads) was readily outcompeted by addition of GFP-Pk to the solution (Fig 4B, quantifed in 4C; see also bottom panel in Fig 4B showing that Pk did not outcompete Dsh binding to the Y-peptide). While these interaction studies are consistent with the co-IPs shown above, the *in vitro* peptide assay displayed the above mentioned caveat, as for example the unphorphorylated peptide was bound equally by both Dsh and Pk, when assayed independently (S4B Fig).

In summary and taken together, the above data are consistent with the model that (i) the Vang region centered around Y374 is necessary and sufficient for binding to Pk and Dsh, (ii) Pk preferentially interacts with this region when Y374 is phosphorylated, and (iii) Dsh requires the structural features of an aromatic ring at Y374 in its binding behavior to Vang. Furthermore, these experiments defined single amino acid mutations, Y374F and V376A, that selectively abrogated binding to either Pk (Y374F) or Dsh (V376A). These observations allow for the first time the functional testing of a direct Vang-Dsh interaction requirement *in vivo*.

### Single point mutants show PCP defects

**Fig 5:**
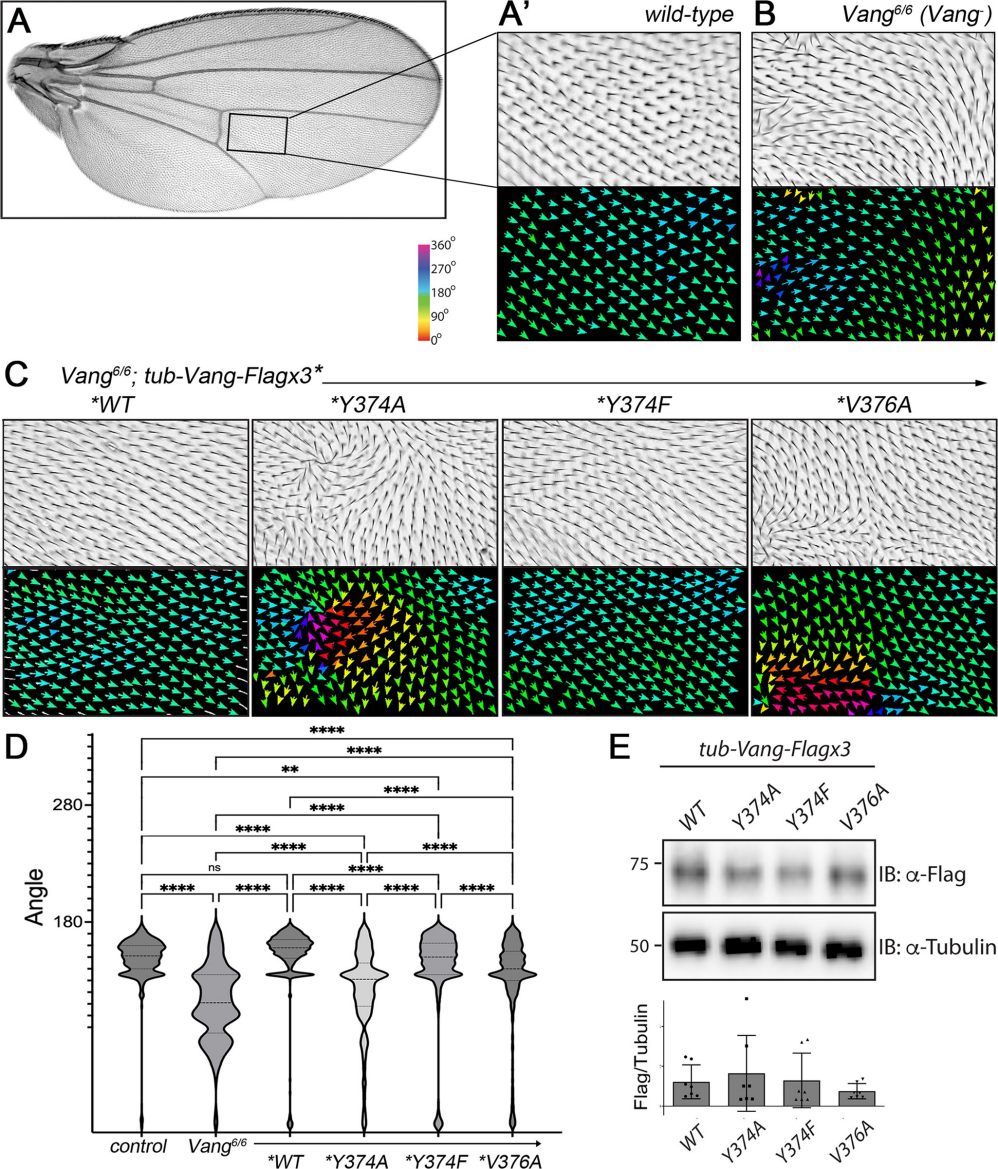
Single Vang point mutants in the Pk and Dsh interaction region display PCP defects *in vivo*. (**A**) *Drosophila* wing (distal is right) and (**A’**) higher magnification of boxed area in wing on left. Cellular orientation is reflected by single hairs pointing distally, also visualized by colored arrows reflecting cellular orientation in lower panel. Angles were determined using plugin FijiWingsPolarity (ref: [54]). (**B**) Image of same region as in **A’** of a *Vang*^*6*^ (null mutant) wing. Note misoriented cellular hairs forming waves and whorls, and schematic refelction of mispolarization by color coded arrows. See colored bar in **A** depicting color scheme. Wild-type (distal) orientation is associated with turquoise in the 160–180° range. (**C**) Sample images of cellular (mis)orientation in adult wings of *Vang*^*6*^ mutant flies (same region as in **A’-B**) upon rescue with *tubulin-*promoter expressed *Vang-Flagx3* transgenes with *WT (wild-type)*, *Y374A*, *Y374F* or *V376A*, respectively as indicated. Angles of cellular orientation are visualized through colored arrows in lower panels (see color bar/scale in panel **A** above). Note that while Vang-*WT* fully rescues the mutant phenotype (and appears like wild-type, compare to **A’**), all single point mutants fail to rescue the mutant defects. Note also that Vang-Y374A most resembles the *Vang* null phenotype, and the other point mutations, display different phenotypes. (**D**) Quantification and statistical analyses of the different behaviors of the individual Vang binding mutants in the *in vivo* rescue assay of the *Vang-/-* null allele, shown as violin plots displaying cellular hair orientation angles for the indicated genotypes, within the ROI area shown in panels A’-C from 3 independent wings were combined. Statitistical analysis was determined with one-way Anova (and Tukey’s post test to compare all samples with each other): **p <0.005, and ****p<0.0001. Note that all genotypes show statitistically different phenotypes from all other gentypes, with the exception of wild-type and the *VangWT* transgene rescue of the *Vang-/-* null allele. (**E**) Western blot of wing disc lysates from the indicated genotypes. Note comparable levels of expression in all transgenes.

To investigate the functional consequences *in vivo* of altering the interaction of Vang with Pk and Dsh, we performed rescue experiments using transgenes carrying the different single point mutants. We expressed Vang-Flagx3 WT, Y374A, Y374F and V376A, using a direct *tubulin*-driven expression in a *Vang* null mutant background (*Vang*^*6*^). The control wild-type construct *tub-VangWT* was able to fully rescue cellular hair orientation in *Drosophila* wings (Fig 5A–5C, see 5D for quantification and statistical analyses; see 5B for *Vang*^*6*^ null mutant control). Each of the single point mutants, Vang-Y374A, Vang-Y374F and Vang-V376A, rescued the *Vang*^*6*^ loss-of-function phenotype partially, displaying varying degrees of cellular hair orientation defects (Fig 5C, quantified in 5D). The phenotypes were quantified using FijiWingsPolarity [54], which revealed phenotypic differences from *VangWT* control in each case, and also among all individual point mutations (compare panels in Fig 5C and quantifications in 5D). Importantly, while the rescue was partial in all three cases, the phenotypes were significantly different from the “non-rescued” null allele and the rescued *VangWT* wings, consistent with the notion that all three point mutants retained partial function as expected. Also, they all displayed different phenotypes, which were visibly and statitistally different from each other (and the null allele; Fig 5C and 5D), suggesting differing partial function, consistent with their distinct biochemical behavior. Consistent with the molecular binding data, Vang-Y374A, which should interfere with direct binding of either Pk or Dsh showed the most severe defects (see cellular hair angle distribution in Fig 5C and 5D), closest to the *Vang* null phenotype but nonetheless different (see statistical analyses in Fig 5D). The point mutations affecting binding to a single effector (Y374F only binding Dsh, and V376A only interacting with Pk) showed a better rescue as compared to Y374A (Fig 5C and 5D; see also Discussion). Of note, the phenotypic differences were not due to different expression levels, as all genotypes showed similar protein levels in larval disc lysates (Fig 5E).

To expand on the different behavior of the individual Vang point mutants *in vivo*, we next tested their effects in a gain-of-function (GOF) assay in developing wings. A classic feature of core PCP factors is that their overexpression also causes PCP defects. Each core factor has thus a reproducible LOF phenotype and also a GOF phenotype, both reflected in stereotypic defects highly similar from wing to wing of any given genotype. As such the Vang-*wt* GOF (via *nubbin-Gal4*) induces similar misorientations from wing to wing (in any specific region of a wing; see wing ROI samples in S5A–S5B Fig; boxed areas in blue and red in S5A Fig correspond to left and right panels in S5B Fig, respectively). As such, Vang overexpression, via *nubbin-Gal4*, of either Vang-*wt* or any Vang-point mutant (see indicated genotypes in S5 Fig), caused cellular orientation defects with wings of the same genotype looking similar to each other, but with different appearances between the distinct Vang-mutant genotypes (S5 Fig). This confirmed that, for example, Vang-Y374F (binding only Dsh) overexpression caused distinct defects from Vang-WT or Vang-YY373DD (binding predominantly Pk) or Vang-Y374A (binding neither factor). Accordingly, all specific Vang mutations tested displayed distinct behavior (S5B Fig, see quantifications in angle distribution graphs on right of each panel; note expression levels of each mutant protein were comparable, S5C Fig).

Together with the rescue data (Fig 5), these results are consistent with the notion that Vang interacts physiologically with both cytoplasmic core PCP factors, Pk and Dsh, during planar polarity establishment *in vivo*. Together with the biochemical dissection, these data thus suggest that Y374 phosphorylation is a critical node for physiological activity of Vang during PCP establishment and for the resolution of core PCP complexes into the Vang and Fz associated “antagonistic polarity domains” (see also Discussion).

### Vang point mutants affect core PCP factor localization during PCP establishment

**Fig 6:**
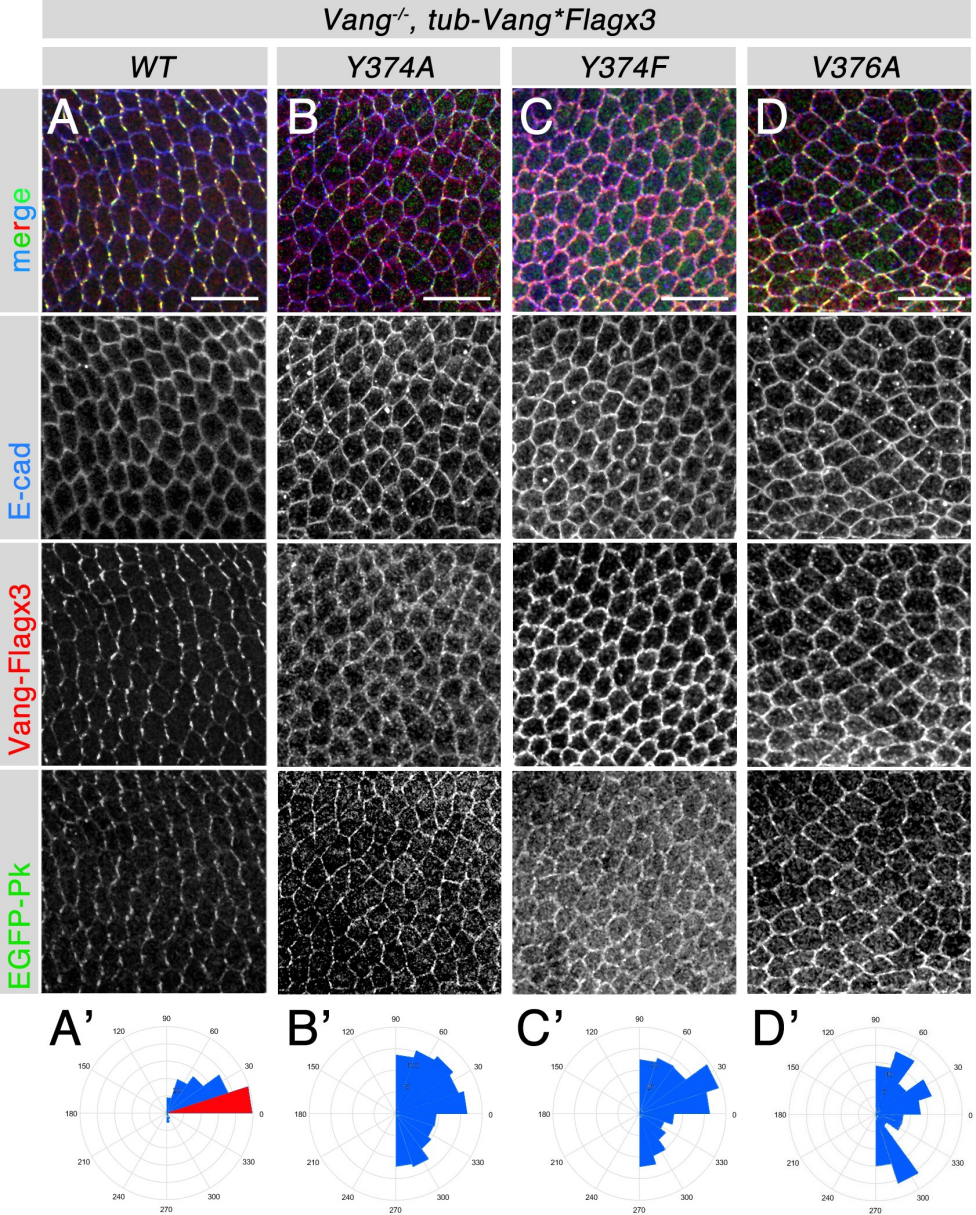
*Vang* point mutants affect localization of core PC factors *in vivo*. (**A-D**) Confocal images of 28-30hr APF pupal wings of the genotypes indicated above panels. All pupal wing tissue is oriented horizinally with proximal side being left and distal right, stained for Vang-Flagx3 (anti-Flag, red), EGFP-Pk (anti-GFP, green), and E-cad (blue). (**A**) *Vang*-/-; *tub-VangWT-Flagx3*, (**B**) *Vang*-/-; *tub-VangY374A-Flagx3*, (**C**) *Vang*-/-; *tub-VangY374F-Flagx3*, (**D**) *Vang*-/-; *tub-VangV376A-Flagx3*. Grayscale single channel micrographs are shown in lower panels as indicated on left side. Note that Pk is still localized to membranes in all three point mutants, albeit less enriched. Scale bars: 20μm. (**A’-D’**) Quantification of polarity angles presented as rosette diagrams of VangFlagx3 protein in the respective genotypes indicated: (A’) *Vang*-/-; *tub-VangWT-Flagx3*, (B’) *Vang*-/-; *tub-VangY374A-Flagx3*, (C’) *Vang*-/-; *tub-VangY374F-Flagx3*, (D’) *Vang*-/-; *tub-VangV376A-Flagx3*. Note that the majority of cell orientation angles is in the proximo-distal axis (horizontal, highlighted in red in rosette diagram) in the *Vang*-/-; *tub-VangWT* background (**A’**, indistinguishable from wild-type), but that cellular orientation is largely randomized in all three point mutants (**B’-D’**, very similar to the Vang null allele). See also S6 Fig for additional core factor stainings and polarity analyses in these genetic backgrounds.

To get further functional insight into how the *Vang* point mutations affect PCP establishment and core PCP factor localization *in vivo*, we next tested the localization of these mutants and associated other core factors. Using the rescue transgenes (with a *tub-Vang-Flagx3* backbone) in a *Vang*^*6*^ null background, we analyzed the localization of Vang-Flagx3 WT, Y374A, Y374F and V376A, Fmi, EGFP-Pk, and DshGFP with E-cad as cellular outline control. The polarity of these proteins was evaluated using the Packing Analyzer suite [55] (Figs 6 and S6). Vang-WT displayed wild-type localization, polarized along the proximo-distal axis (Fig 6A) with all other core PCP factors tested, Fmi, Pk, and Dsh, also showing wild-type polarized, membrane associated distribution within the proximo-distal axis in pupal wing tissue at 28h APF (Figs 6A and S6A). In contrast all three point mutants displayed largely apolar (or mispolarized) distribution (Fig 6B–6D, mispolarization is highlighted in the angle quantification as seen in the rosette diagrams in panels 6A’-6D’). Apolar PCP core factor distribution was also evident in analyses of Fmi distribution (S6 Fig). Pk and Dsh protein distribution was also apolar (Figs 6B–6D and S6B–S6D, see also below). However, both cytoplasmic core PCP factors, Dsh and Pk, maintained significant membrane association in all point mutant backgrounds (Figs 6B–6D and S6B–S6D, see also below). All three Vang point mutants appeared membrane associated similar to Vang-WT (Fig 6), suggesting that Vang trafficking is not affected in these point mutants (see Discussion). While it is possible that differences between the individual Vang point mutants exist, also based on past observations for example [56], a carefull side-by-side comparison of the respective point mutations would be needed. Importantly, none of the point mutations caused a complete loss of membrane associated Pk or Dsh. This is consistent with previous data demonstrating that even in *Vang* null clones Pk is still partially retained at cellular membranes, likely due to being farnesylated and requiring this modification for function [12,48].

In parallel to *in vivo* localization studies, we also tested whether Vang/Vangl variants might differently affect the stability of core PCP membrane complexes, which are anchored at cell junctions by the atypical cadherin Fmi (Celsr1 in mice). To do this, we transfected mouse primary keratinocytes with Celsr1-GFP and mCherry-Vangl2, which co-localize to the junctional interfaces between expressing cell pairs mediated by homotypic adhesion of Celsr1 (S7A Fig) [14]. We then used fluorescence recovery after photocbleaching (FRAP) to determine the mobility of Celsr1 and Vangl2 proteins located within the junctional membrane. Compared to Celsr1 and Vangl2 proteins located at free edges of the cell, Celsr1 and Vangl2 were largely immobile at cell junctions (~80% immobile fraction)(S7B–S7C Fig), as previously shown [36,57]. Mutation of Vangl2 Y308 to A (equivalent to *Drosophila* VangY374A) did not affect its ability to localize to cell junctions (S7A Fig), nor did the mutation impact Vangl2 mobility at cell junctions, or that of co-expressed Celsr1-GFP (S7B–S7C Fig). We conclude that although the Y308 residue in Vangl2 can be phosphorylated in mammalian epidermal cells, it is not required for Vangl2 recruitment to or stabilization at cell junctions.

Taken together, the *in vivo* analyses of the Vang point mutations confirm that the direct interactions with Pk and Dsh are critical for correct PCP establishment and polarized core PCP complex segregation and thus Vang function. However, they also confirmed that Vang is not essential for membrane recruitment of either cytoplasmic PCP factor and also that the phosphorylation status of its Y374 residue (Y308 in mice) has no or minimal impact on junctional PCP core complex stability (see Discussion).

## Discussion

Here we identify through mass spectrometry analyses with mouse skin epidermis samples phosphorylation on mouse Vangl2 Y308 residue (equivalent to Y374 in *Drosophila* Vang). This tyrosine lies within with the overlapping binding regions of Pk and Dsh in Vang/Vangl, and, importantly, its charge/phosphorylation status regulates selective binding between Pk and Dsh, with phosphorylation tipping the balance towards Pk binding. We demonstrate *in vivo* that binding of Vang to both cytoplasmic core PCP factors is physiologically important (which is the first *in vivo* evidence for a Vang-Dsh binding requirement). Our study provides novel insight into the critical importance of Vang tyrosine phosphorylation and reveals mechanistic features of how regulation of the binding of antagonistic PCP factors to Vang/Vangl during the process of PCP complex segregation and polarity establishment is achieved.

### Phosphorylation of Y374/Y308 at the center of Vang/Vangl interactions with Pk and Dsh

While previous work defined a broad region within the C-tail of Vang to interact with both Pk and Dsh [12,53,58–60], the mechanistic regulation and physiological significance of these interactions remained unresolved. Importantly, the defined region is conserved between *Drosophila Vang* and mammalian *Vangl1/2* genes. Our data reveal that a small conserved stretch of amino acids within this broader region is both necessary (as shown in the whole Vang protein) and sufficient (as deduced from the *in vitro* peptide assays) to interact with both cytoplasmic core PCP factors. This region is well conserved between all Vang family members and centered on the tyrosine, which can be phosphorylated, as our mass spec data reveal. Mutational studies define that Pk binding is mediated by tyrosine phosphorylation and associated negative charge, while Dsh requires the aromatic ring found in tyrosine (and also phenylalanine) for its binding to Vang. It is worth noting that this Vang region, shared by both Pk and Dsh for binding, is specific for these two factors, as other Vang associated cytoplasmic PCP proteins, for example, Dgo and Scrib, are not affected by mutations within this domain.

The importance of the Vang-Pk complex has been well documented *in vivo* and is also the core of one of the two stable PCP “core complexes” that result from PCP factor interactions and signaling (reviewed in [1–7]). In contrast, an interaction between Vang/Vangl and Dsh/Dvl family members has only been documented biochemically [12,53,58–60]. The dissection of binding requirements allowed us to generate single point mutations in Vang that separate binding to only one of the cytoplasmic factors, either Pk or Dsh. The associated *in vivo* rescue experiments provided the possibility for physiological testing of a functional requirement of the individual interactions between Vang and Dsh or Pk. While all three point mutations display partial rescue, their function is reduced and thus the respective amino acids are physiologically required. Interfering with binding of Vang to both factors (VangY374A) shows the weakest rescue, with the mutant displaying defects that are more similar to the *Vang*^*-*^ null phenotype than the other point mutants. Nevertheless, as the point mutations affecting individual Vang-Pk or Vang-Dsh interactions also displayed only partial rescue of the *Vang*^-/-^ defects, these data indicate that interactions with either cytoplasmic PCP factor, Pk and Dsh, are critical for *in vivo* Vang function in PCP core complex localization and hence PCP establishment. The phenotypic defects with the Vang-V376A mutant seen in adult wings suggest that it might behave as a mild neomorph (see Fig 5C and 5D), although no dominant effect was observed when heterozygous over Vang-WT. Importantly, this is the first physiological *in vivo* evidence demonstrating that Vang has a requirement to interact with Dsh during PCP complex segregation. Of all point mutants tested, Vang-Y374F, showed the strongest partial rescue (appeared closest to wild-type). This is consistent with the notion that Pk does not strictly require binding to Vang for membrane association [12,48], and that formation of a Vang-Pk complex is much more complcated than a single interaction between the two proteins. Of note, each single point mutant affects the formation of the stable polarized core PCP complexes, as evident in the protein localization studies in 28h APF pupal wings, suggesting that interfering with any interaction among the core PCP factors causes a significant disruption to the PCP interaction cascade and network, needed for normal asymmetric complex polarizations.

### How does Vang Y374/Y308 phosphorylation affect PCP complex resolution?

It is intriguing to think about how phosphorylation, and lack thereof, affects the formation of stable core PCP complexes. Our data indicate that binding of Dsh/Dvl to Vang/Vangl is physiological, and yet in standard co-staining studies Vang and Dsh do not co-localize. How does Dsh binding to an unphosphorylated Y374 region affect core PCP complex formation?

There are a few potential scenarios, and importantly Vang is not a major membrane recruiter of Pk, if at all [12,48] [and this work] and thus other factors likely contribute to this. First, a Vang-Pk association, which we assume is stable in wing cells in the proximal junctional membrane region, should likely not form in other areas of the cell membrane. As such a Vang-Dsh transient/intermediary interaction might serve a function to prevent Vang-Pk binding. If the kinase in question is asymmetrically localized or active, for example in the proximal area, then—and only then—a switch from Vang-Dsh to a Vang-Pk interaction would occur. If the kinase in question is not asymmetrically active or localized, Dsh binding to this Vang region might be required to prevent the kinase to act on Vang in cell membrane domains, where formation of a Vang-Pk complex should not form, for example the distal vertex of a wing cell. In such a mechanistic scenario Dsh would keep Vang/Vangl “flexible” to find the right cellular context, where/when the presence of the kinase would initiate the switch to a Vang-P at Y374 (Y308 in mVangl2) and thus support an interaction with Pk and its local effectors. While these are intriguing mechanistic models, they remain speculative.

In general, the function of Vang/Vangl proteins in PCP establishment remains unresolved. While Vang family proteins are critical for the process and they can physically interact with all other core PCP factors, their contribution to the stability of the intercellular junctional complexes remains unclear, which seem to mainly require Fz-Fmi::Fmi interactions [34,61], although Vang/Vangl proteins are part of these asymmetric complexes and bind to Fz intercellularly [35]. Moreover, the non-stoichiometric manner in which the stable PCP complexes form [62], with for example one single Pk molecule per 6 Vang molecules, suggests complicated mechanistic scenarios that do not rely on one-to-one protein interactions. Importantly, the formation and maintenance of stable PCP complexes requires also Dgo [33] (Diversin in vertebrates; [63]) and extends beyond interactions among the core factors, including Scribble [31,32] and CK1ε [45–47,49] and many regulatory interactions are still to be discovered (see below). Complex *in vivo* experiments will be necessary to better understand the mechanistic sequence of events.

### What is the tyrosine kinase acting on Vang/Vangl?

It is currently unclear which tyrosine kinase(s) act on Vang to mediate its phosphorylation on the Y374 residue (Y308 in mVangl2) and this is one of the regulatory interactions to be still discovered. Sequence motif searches in the Vang Y374 flaking region suggest that Src family kinases could be involved, with no other kinase family having a higher probability (by sequence alignment searches). It is however technically difficult to prove that Src kinases indeed act on this Vang residue in a physiological context (see below), and unfortunately *in vitro* kinase assays have proven uninformative, as most tyrosine kinases tested could phosphorylate Vang in such assays on multiple residues. Redundancy of Src kinases is an issue in *in vivo* studies in both our systems, mouse skin and *Drosophila* wing epithelia, as there are several Src family kinases in both *Drosophila* and mice, for example [64–68]. Moreover, in addition to cell survival requirements, many cellular functions are associated with Src family kinases (rev in e.g. [69–71]. For example, in *Drosophila* the two main Src family members are either viable with no overt developmental phenotypes in imaginal discs (Src64, redundant with Src42) or are largely cell lethal (Src42) when analyzed *in vivo* [64,68]. They have also been linked to a vast variety of cellular functions, ranging from cytoskeletal regulation and cell adhesion, to synaptic plasticity, proliferation, cell death, and others (reviewed in [69–71]. Src kinases remain nonetheless likely candidate(s), as we (i) observe GOF phenotypes consistent with a PCP function and (ii) genetic interactions with these Src GOF defects suggest that Vang is required in these contexts. However, again, a loss-of-function scenario to really demonstrate a Src function in PCP establishment remains elusive and should be the focus of future studies.

## Materials and methods

### Mouse lines and embryonic skin harvesting

#### Ethics statement

All mouse work in this study was approved by Princeton University’s Institutional Animal Care and Use Committee (IACUC) under protocol number 1867.

K14-GFP-Vangl2 transgenic mice (FVB background, [52] were housed in an AAALAC-accredited facility following the Guide for the Care and Use of Laboratory Animals. E15.5 embryos were harvested from K14-Vangl2-GFP heterozygous dams in cold PBS and screened for GFP expression using a stereomicroscope equipped with epifluorescence. Full thickness backskins were dissected from both GFP+ and GFP- littermates embryos and flash frozen immediately in liquid nitrogen (LN2). LN2 was removed by evaporation and frozen backskins were stored for up to 3 months at -80C until cryolysis.

#### Epidermal cryolysis and immunoprecipitation of GFP-Vangl2

Frozen skin samples pooled from four (for IP-Western) or eight (for IP-MS) GFP-Vangl2 and control backskins were processed via cryolysis using a CryoMill (Retch). Briefly, 2ml LN2-frozen lysis buffer droplets (Tissue Extraction Buffer, 1% Triton X-100, 10mM EDTA, 0.3mg/ml PMSF plus protease inhibitors in PBS) were mixed with frozen skin samples and processed by cryogenic grinding for 20 min using a ball mill cooled with LN2. Finely ground frozen epidermal-lysis buffer mixtures were lysed by thawing on ice for 1.5-2hrs. Lysates were cleared by adding 50ul Pansorbin and centrifuging at 14K rpm for 10min.

Pre-cleared lysates were transferred to pre-washed, αGFP (rabbit αGFP, AbCam) antibody bound beads and incubated while rotating for 3 hours at 4 degrees C. Immunoprecipitates were washed twice with lysis buffer and eluted with 40ul 2X SDS sample buffer. For western blotting, 40ul total lysate and 40ul IP were run on a 10% SDS-PAGE gel and transferred to a PDVF membrane, blocked for 1hr at room temperature, incubated with primary, chicken αGFP (1:5000, Abcam) overnight at 4°C. After several washes in PBS-T, membrane was incubated for 45 min at room temperature with HRP α-chicken secondaries (1:2500), developed with BioRad Clarity ECL reagent and imaged on both film and with BioRad imager.

For IP-MS samples, 20ul total lysate and 40ul IP were run on a 7.5% SDS-PAGE gel. Gel was fixed in 50% MeOH + 7% Acetic Acid for 30–60 minutes, rinsed with H_2_0, stained with SPYRO Ruby overnight, and washed twice for 5 min each in 10% MeOH + 7% Acetic Acid. Gel imaging was performed on a Typhoon FLA-7000 (GE Healthcare).

#### Proteomic sample preparation and mass spectrometry

IP Bands at ~85-90KD were excised then diced, and subjected to in-gel thiol reduction/alkylation and trypsin digestion using a method adapted from Shevchenko et al [72] to process samples for LC-MS/MS. Briefly, gel cubes were destained and washed extensively in 100 mM ammonium bicarbonate buffer, pH 8.8 (ABC), treated with 50 mM TCEP in ABC for 1 h at 55°C, washed, subjected to alkylation with 55 mM iodoacetamide in ABC for 30 min at room temperature in the dark, washed, and finally digested overnight with 1 ug Promega Trypsin Gold (Promega) per gel slice. Peptides from in-gel digest eluates were desalted using STAGE-Tips 9 prior to LC-MS analyses.

LC-MS/MS analyses were performed on a high-resolution, high-mass-accuracy, reversed-phase nano-UPLC-MS platform, consisting of an Easy nLC Ultra 1000 nano-UPLC system coupled to an Orbi Elite mass spectrometer (ThermoFisher Scientific) equipped with a Flex Ion source (Proxeon Biosystems, Odense, Denmark). LC was conducted using a trapping capillary column (150 μm x ca. 40 mm, packed with 3 μm, 100 Å Magic AQ C18 resin, Michrom, Auburn, CA) at a flow rate of 5 μL/min for 4 min, followed by an analytical capillary column (75 μm x ca. 45 cm, packed with 3 μm, 100 Å Magic AQ C18 resin, Michrom) under a linear gradient of A and B solutions (solution A: 3% acetonitrile/ 0.1% formic acid; solution B: 97% acetonitrile/ 0.1% formic acid) from 5%-35% B over 90 at a flow rate of 300 nL/min. Nanospray was achieved using Picospray tips (New Objective, Woburn, MA) at a voltage of 2.4 kV, with the Elite heated capillary at 275°C. Full-scan (m/z 335–1800) positive-ion mass spectra were acquired in the Orbitrap at a resolution setting of 120,000. MS/MS spectra were simultaneously acquired using CID in the LTQ for the fifteen most abundant multiply charged species in the full-scan spectra, having signal intensities of >1000 NL. To aid in phosphosite mapping, Vangl2-positive slices were subjected LC-MS/MS over a 180 min gradient using the CID parameters above and also a second round of LC-MS/MS over a 180 min gradient during which MS/MS spectra were acquired by multistage activation (MSA) for the top 10 most abundant ions in the full-scan spectra, using excitation at the precursor *m/z* value as well as those corresponding to the neutral losses of phosphonic and phosphoric acids for ions of charge +2 and +3. Lockmass was employed, maintaining calibration to 2–3 ppm of accurate mass.

#### Mass spectrometric data analysis

Resultant LC-MS/MS raw data files were processed using ProteomeDiscoverer (v. 1.4, ThermoFisher), to match MS/MS spectra against the UniProt *Mus musculus* database, or a GFP-Vangl2 fusion protein construct subset database using the Mascot search engine (v. 2.4, Matrix Science, London, UK.), allowing for a parent ion mass window of ±6 ppm, ≤ 3 missed trypsin cleavages, serine, threonine and tyrosine phosphorylation, methionine oxidation, asparagine and glutamine deamination and *N*-terminal protein acetylation as variable modifications, and carbamidomethylation of cysteines as a fixed modification. Peptide assignment cut-offs were specified at a high confidence level (<1% FDR). Phosphosite localization confidence scoring was achieved using the PhosphoRS 9 [73] (v. 3.1) node within the ProteomeDiscoverer framework. Relative abundance levels for proteins between experimental and controls were estimated using spectral counting. Raw mass spectra were visualized using Xcalibur (v. 2.2, ThermoFisher) and peptide spectral matches were visualized using ProteomeDiscoverer or Scaffold (v. 4.3.4, Proteome Software, Portland, OR). All phosphopeptide assignments were further validated by manual inspection.

#### DNA constructs and S2 culture

Constructs used were as follows; pAc5.1-Flagx3, pAc5.1-Myc-Pk, pAc5.1-Dsh-GFP, pAc5.1-Scrib-PDZ-3-4-HA and pAc5.1-HA-Dgo (all gifts from Dr. Jenny, AECOM, USA) and pAc5.1-Vang-Flagx3 [44]. GFP-TZ was used as a control, and is the *Drosophila* ciliary protein Mks1. pAc5.1-Vang-Flagx3-Y374A, FKYY371AAYA, Y374F and V376A were generated though site-directed mutagenesis. C-terminal truncations of Vang were generated through PCR and cloned into pAc5.1-Flagx3 using NotI-XbaI restriction sites. All primers used are available upon request.

Unless otherwise stated, lysates were prepared from S2 cells. S2 cells were maintained according to standard protocols, and were grown in Schneider’s Medium (Gibco) supplemented with 10% heat-inactivated Fetal Bovine Serum (Gibco). Cells were plated in 12 well plates at a dilution of 1.5x10^6^ and were transfected with the indicated constructs using Effectene (Qiagen) according to manufacturer’s protocols. Cells were lysed ~48 hrs later in buffer containing 50mM Tris-HCl pH 7.5, 150mM NaCl, 1mM EDTA and 1% Triton-X-100.

#### Pull-downs, immunoblotting and peptide interactions

For Flag pull-down experiments, lysates were extracted from S2 cells or from larval wing discs and were incubated at 4°C overnight with 10μl anti-Flag M2 affinity gel per sample. Washes were performed in buffer containing 50mM Tris-HCl pH 7.5, 350mM NaCl, 1mM EDTA, 0.1% SDS before elution in final sample buffer. For peptide binding, Vang peptides were conjugated to beads to enable assessment of direct binding. Lysates were generated in S2 cells, and the lysate was divided equally between tubes containing either 20μl of conjugated phospho-peptide or the unphosphorylated form and incubated at 4°C overnight. Washes were performed in buffer containing 10mM Tris-HCl pH7.5, 350mM NaCl, and 0.5mM EDTA before elution in final sample buffer. For GFP pull-downs. Samples were resolved by polyacrylamide gel electrophoresis according to standard protocols.

The following primary antibodies were used for immunoblotting; Flag (Sigma M2 1:5000), Gamma-tubulin (Sigma GTU-88 1:1000), GFP (Roche 7.1&13.1 1:1000), Myc (SCBT 9E10 1:1000), Phospho-tyrosine (Millipore PY20 1:1000).

#### *Drosophila* strains, dissections and phenotypic analyses

Flies were raised on standard medium, and maintained at 25°C unless otherwise stated. To generate *UAS-Vang-Flagx3* mutant transgenic flies, site-directed mutagenesis was performed on vector pUAST-Vang-Flagx3-attB [44], before insertion into BDSC stock number 9750. To generate *tub-Vang-Flagx3* mutant flies, site-directed mutagenesis was performed on vector pCaSpeR-tub-Vang-Flagx3 [47]. All transgenic strains were generated via Bestgene Inc.

Wing discs were dissected from third instar larvae and prepared through incubation in final sample buffer at 95°C. For phosphatase treatment, wing discs were collected in PBS and transferred to lysis buffer containing 50mM Tris-HCl pH 7.5, 150mM NaCl, 1mM EDTA, and 1% Triton-X. Lysates were then incubated at 30°C for 30 minutes with lambda protein phosphatase (NEB).

Adult wings were collected in PBS containing 0.1% Triton-X-100 (PBST) and incubated for 1 hr at room temperature before mounting in 80% glycerol in PBS. Hair orientation was quantified using the FijiWingsPolarity plugin [54].

#### Western blot quantification and statistical analyses

Western blot quantifications were made in ImageJ, measuring the intensity of each band and substracting the background. Each measurement was normalized to the loading control(s). To determine statistical differences between data sets of continuous data, we performed nonparametric ANOVA and Tukey’s post test in GraphPad. *P*-values lower than 0.05 were considered significant, and levels of significance are indicated by number of asterisks (see Figure legends for specific details). The error bars represent the SEM.

#### *Drosophila* pupal wing fluorescence stainings and confocal analyses

White pupae were collected (0h APF/after puparium formation) and aged at 25°C until dissection at 28-30h APF. Dissections were performed as follows: in brief, pupae were immobilized on double-sided tape, removed from the pupal case, and placed into PBS, in which pupae were partially dissected to remove fat tissue, fixed in 4% paraformaldehyde in PBS for 45 min at RT or overnight at 4°C, and washed 3x in PBS and 0.1% Triton X-100. Wing membranes were removed, and immunostaining was performed by standard techniques. In brief, tissue was incubated in wash buffer containing 10% normal goat serum overnight for primary antibody (4°C), washed 3x with PBS, and incubated with the secondary antibody for at least 2 h (25°C) and fluorescent phalloidin for staining the actin cytoskeleton. Wings were washed 3x with PBS and mounted in Mowiol (see also [74] for more details). Pupal-wing images were acquired at RT using a confocal microscope, Leica SP8. Images were processed with ImageJ (National Institute of Health) and analyzed using the Packing Analyzed software Fiji plug-in [55].

#### Fluorescence recovery after photobleaching (FRAP) analysis

Approximately 150,000 wild type CD1 keratinocytes were seeded in no.1.5 glass bottom dishes (ibidi #81151) coated with fibronectin. 20–24 hours post-plating, cells were co-transfected with Celsr1-GFP and mCherry-Vangl2 or Celsr1-GFP and mCherry-Vangl2^Y308A^ plasmids. 24 hours after transfection, cells were switched to E-media containing 1.5mM Ca2+ and incubated for an additional 20–24 hours for adequate Celsr1-GFP expression and keratinocytes to form stable cell-cell junctions. Before imaging, cells were switched to phenol-red free E-media with 1.5mM Ca2+. Cells were imaged using a 488nm and 561nm laser, 60X magnification objective (with additional zoom that rendered a pixel size of 110nm) on Nikon A1R-STED confocal microscope equipped with a stage-top Tokai-Hit incubation chamber to maintain 37 degrees and 5% CO2. Keeping magnification, laser power (both for bleach and acquisition), pixel dwell time and acquisition rate constant across all measurements, 1um diameter circular bleach ROIs and one ROI per junction(s) or cells edge(s) were created for bleaching and recovery. The FRAP acquisition sequence consisted of 3 reference pre-bleach images followed by bleach using 405nm laser and finally 70 frames with 4 seconds intervals to monitor fluorescence recovery at junctions. The acquired images in the time series were checked for Z-drift and corrected for presence of any XY drift in Fiji. A reference ROI was made in a non-bleached region to correct for overall bleaching during image acquisition. A background ROI was created outside the fluorescent cell in each image. The ROI values were extracted from drift corrected images in NIS elements software and subsequently processed in Microsoft excel and Graphpad Prism. Each image time series was background and bleach corrected (to be referred as corrected intensity henceforth) and thereafter the corrected intensity profile was normalized as (F_t_−F_bleach_)/ (F_ini_−F_bleach_), where, F_t_ is the corrected intensity of the ROI at a given time point, F_bleach_ is the corrected intensity at the time point immediately after bleaching, F_ini_ is the mean ROI intensity of the three pre-bleach frames. Each mean recovery curve was fitted to exponential one phase association equation in Graphpad Prism and the fitted Plateau and Y_0_ values were used to determine the immobile fraction = 1- {(Plateau-Y_0_)/(1- Y_0_)}. The averaged traces for each condition was fitted to the model with an r-squared value > 0.9. Data represented is pooled from four independent experiments.

#### Cell culture and transfection

Primary wild type CD1 Keratinocytes were cultured using previously published protocol [75]. Keratinocytes were in E-Media prepared in the laboratory (see [75] for composition) supplemented with 50μM Calcium Chloride. For FRAP experiments, phenol-red free DMEM and HF-12 were used to prepare pigment-free imaging E-media and supplemented with 1.5mM Calcium Chloride. Cells were transfected using Effectene reagent following a modified manufacturer’s protocol. 400ng DNA comprising of Celsr1-GFP and mCherry-Vangl2 [14] or mCherry-Vangl2^Y308A^ (this paper) in a ratio of 2:1 was used for co-transfection.

## Supporting information

S1 Fig IP-MS approach from mouse skin to identify Vangl2 PTMs. (DOCX)

S2 Fig Specificity of Pk and Dsh binding to the Vang region 364–387. (DOCX)

S3 Fig Charged amino acids interfere with Dsh binding to Vang. (DOCX)

S4 Fig Y374 peptide and phospho-peptide binding to Pk and Dsh. (DOCX)

S5 Fig Distinct gain-of-function in vivo behavior of Vang and the respective Pk and Dsh binding mutants. (DOCX)

S6 Fig Vang point mutants affect localization of core PC factors in vivo. (DOCX)

S7 Fig FRAP analysis of junctional Celsr1 and Vangl2 in cultured keratinocytes. (DOCX)

S1 Table Vangl2 peptides and PTMs. (XLSX)
