# Supplementary material for: A Van Gogh/Vangl tyrosine phosphorylation switch regulates its interaction with core Planar Cell Polarity factors Prickle and Dishevelled
Source: PLoS Genet. 2023 Jul 18;19(7):e1010849. doi: 10.1371/journal.pgen.1010849 (PMC10381084; doi:10.1371/journal.pgen.1010849)
Supplement: S1 Fig — (DOCX) [file pgen.1010849.s001.docx]

**
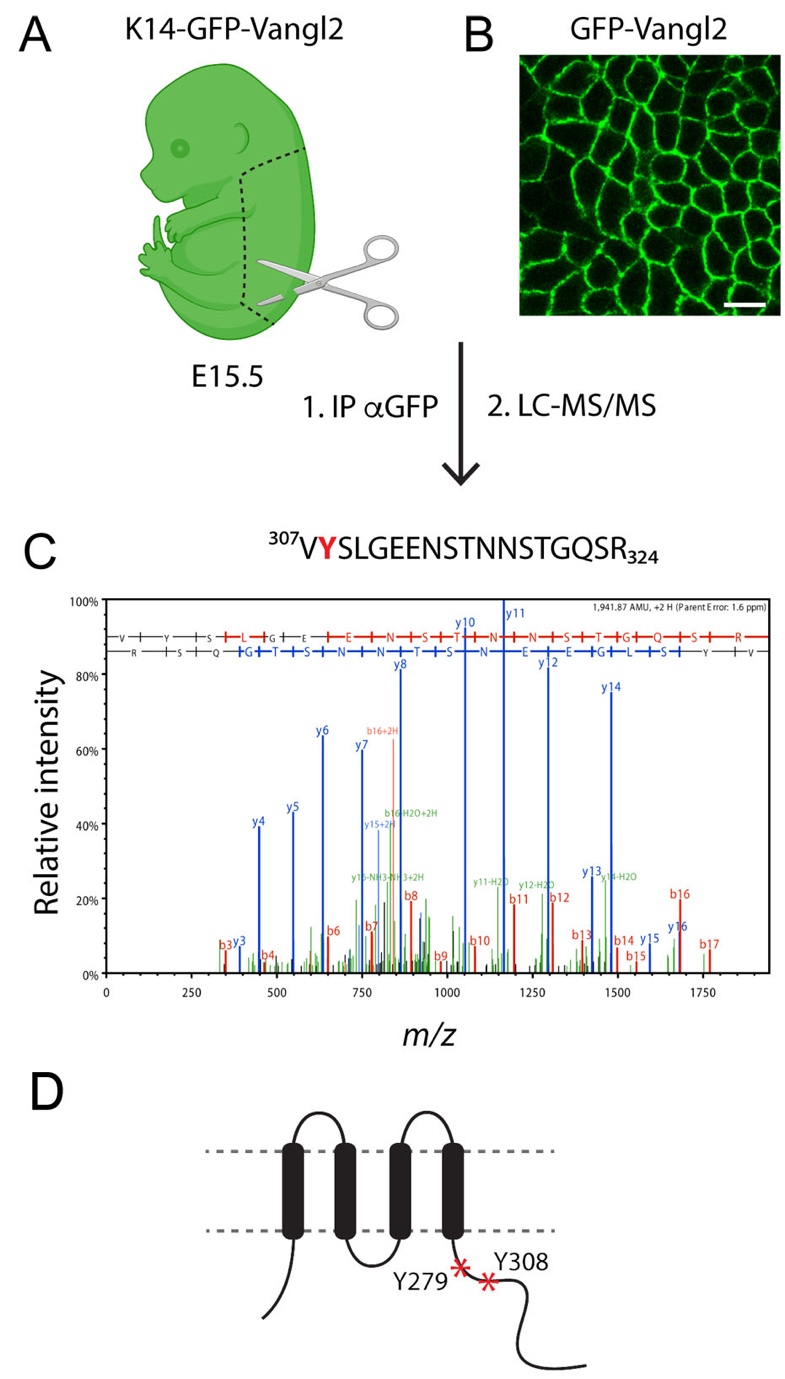
**

**S1 Figure (Supplement to Figure 1):**

**IP-MS approach from mouse skin to identify Vangl2 PTMs.**

**A**. Skins were dissected from E15.5 embryo expressing GFP-Vangl2 under the K14 skin-specific promoter. Epidermal lysates were prepared from frozen and cryo-milled skin samples and GFP-Vangl2 immunoprecipitated using anti-GFP antibodies. Created with BioRender.com

**B.** Planar view of basal layer from K14-GFP-Vangl2 whole mount epidermis. GFP-Vangl2 is localized to cell junctions. Scale bar 10um.

**C.** Representative m/z spectrum of Y308-contining peptide (aa307-324).

**D.** Schematic of mouse Vangl2 protein with asterisks indicating positions of phosphorylated tyrosines Y279 and Y308.
