# Supplementary material for: A Van Gogh/Vangl tyrosine phosphorylation switch regulates its interaction with core Planar Cell Polarity factors Prickle and Dishevelled
Source: PLoS Genet. 2023 Jul 18;19(7):e1010849. doi: 10.1371/journal.pgen.1010849 (PMC10381084; doi:10.1371/journal.pgen.1010849)
Supplement: S2 Fig — (DOCX) [file pgen.1010849.s002.docx]

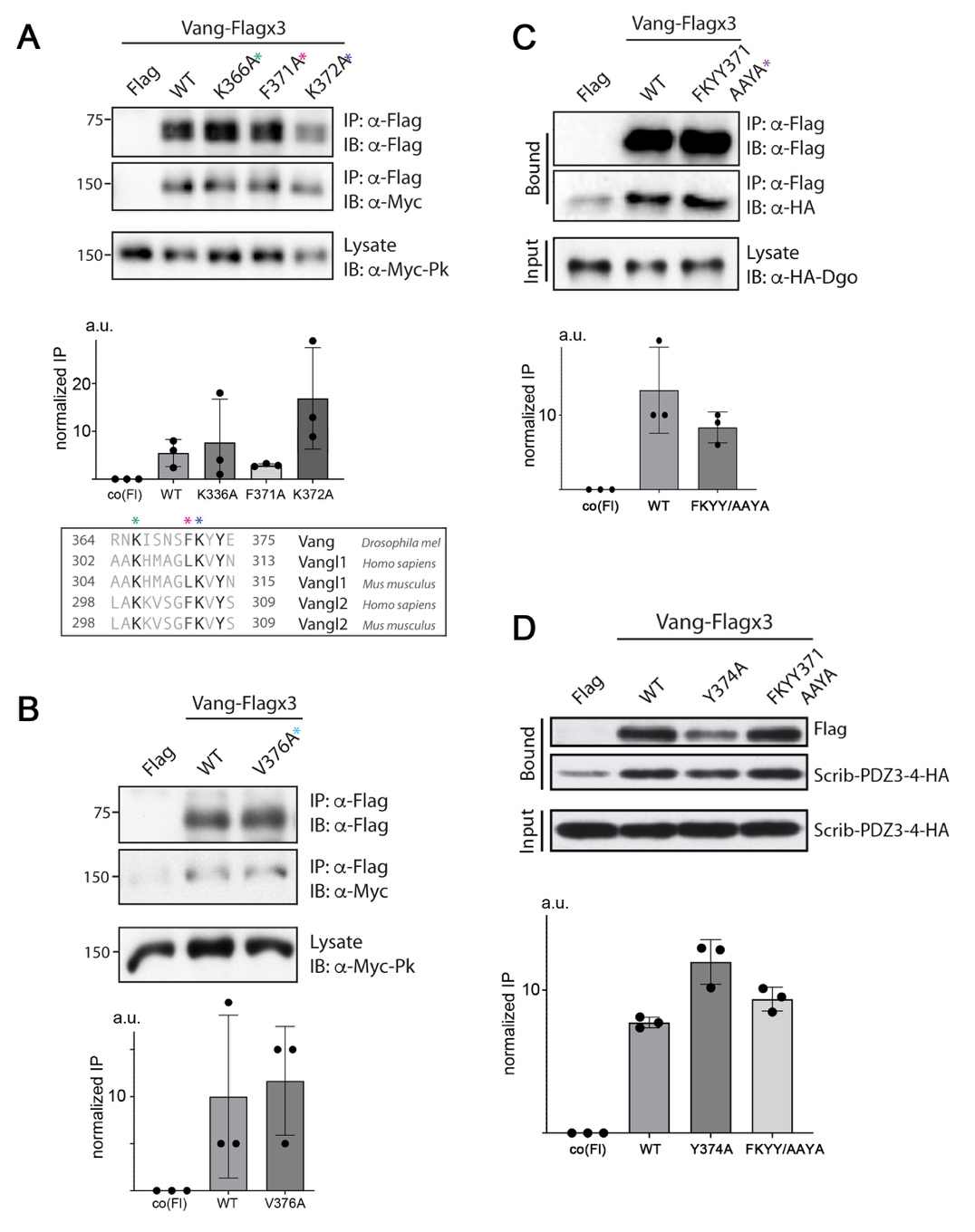


**S2 Figure (Supplement to Figure 2):**

**Specificity of Pk and Dsh binding to the Vang region 364-387**

To further define the binding site, we focused on the conservation within this region, as the mammalian orthologues, VANGL1/2 and PK family members also physically interact. (**A**) A series of point mutations, substituting each conserved or partially conserved residue for alanine was generated and these experiments revealed that substitutions at K366, F371, and K372 had little effect on their own: Western blot showing binding between Myc-Pk and the Vang-Flagx3 point mutants as indicated (see also sequence alignment below in this panel, with colored asterisks highlighting the different amino acids mutated in the experiment). Middle panel shows quantification analysis of binding. All IP results were quantified (shown as arbitrary units) after being normalized to the input of both the Vang protein isoforms and Pk (here and in all other panels of this figure, quantification was performed on three independent replicates). Note that Dsh binding (as Dsh-GFP) was also retained in each of these mutants.

(**B**) Western blot showing binding between Myc-Pk and Vang-Flagx3 V376A point mutant, which abrogates Vang-Dsh binding (see main Figure 2H,H’ for comparison; see main Figure 2E for sequence alignment). Note that Pk binding is retained in the V376A mutants. Bottom panel shows quantification analysis of binding (quantified as outlined in panel **A** with blots from three independent replicates).

(**C**) and (**D**) Control binding experiments for Vang with additional PCP core factors/effectors, Dgo (**C**) and Scribble (Scrib) (**D**), demonstrating that neither Dgo nor Scrib bind to the region where Pk and Dsh interact. Note that mutants in the 371-FKYY-374 motif do not affect the Vang-Dgo and Vang-Scrib interactions. Bottom panels show quantification analyses of the respective binding (quantified as outlined in panel **A**, all experiments were performed in three independent replicates).
