# Supplementary material for: A Van Gogh/Vangl tyrosine phosphorylation switch regulates its interaction with core Planar Cell Polarity factors Prickle and Dishevelled
Source: PLoS Genet. 2023 Jul 18;19(7):e1010849. doi: 10.1371/journal.pgen.1010849 (PMC10381084; doi:10.1371/journal.pgen.1010849)
Supplement: S3 Fig — (DOCX) [file pgen.1010849.s003.docx]

**
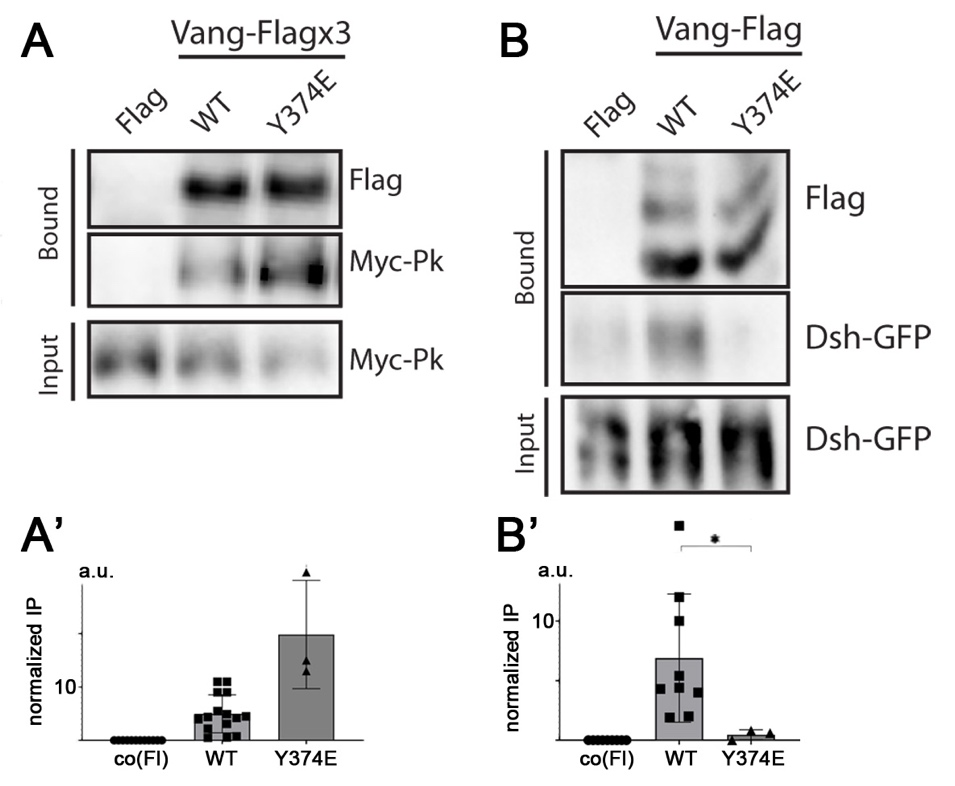
**

**S3 Figure (Supplement to Figure 3):**

**Charged amino acids interfere with Dsh binding to Vang.**

(**A-B’**) Western blots showing binding between Vang-WT (control) and VangY374E, replacing the Y-residue with a charged amino acid. (**A-A’**) Pk binding is not affected (or possibly even increased), in the VangY374E mutant as compared to Vang-WT. (**A’**) Quantification of binding. IP results were quantified (shown as arbitrary units) after being normalized to the input of both the Vang protein isoforms and Pk or Dsh (here and in all other panels of this figure), data was combined from three independent replicates.

(**B-B’**) Dsh-GFP binding is blocked in the VangY374E mutant, replacing Y with a charged amino acid. (**B’**) Quantification and statistical analysis of binding differences. **p<*0.05 as determined with Anova. Note almost complete loss of Dsh interaction with Vang in the mutant Y374E form, as compared to Vang-WT control.
