# Supplementary material for: A Van Gogh/Vangl tyrosine phosphorylation switch regulates its interaction with core Planar Cell Polarity factors Prickle and Dishevelled
Source: PLoS Genet. 2023 Jul 18;19(7):e1010849. doi: 10.1371/journal.pgen.1010849 (PMC10381084; doi:10.1371/journal.pgen.1010849)
Supplement: S4 Fig — (DOCX) [file pgen.1010849.s004.docx]

**
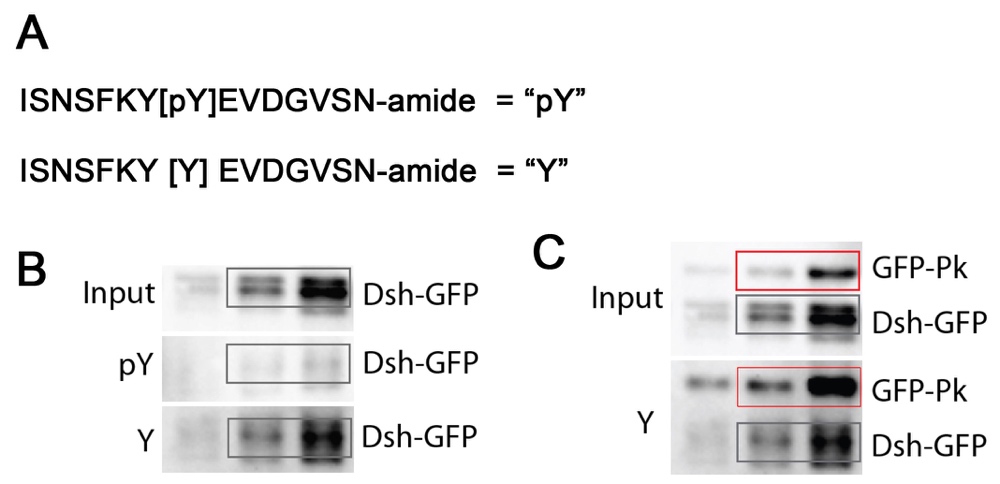
**

**S4 Figure (Supplement to Figure 4):**

**Y374 peptide and phospho-peptide binding to Pk and Dsh.**

(**A**) Sequence of the synthetic peptides used [ISNSFKY[*pY*]EVDGVSN-amide] and the equivalent non-phosphorylated control, with the phospho-peptide denoted as “pY” and the control peptide as “Y” (see also main Figure 4). (**B**) and **C**) While Dsh preferentially interacts with the non-phosphorylated “Y”-peptide. Compare bands in grey boxes between input and pY and Y-bound blots (**B**), Pk can in this strictly in vitro assay bind both peptides, as evident in its interaction with the “Y”-peptide (**C**, red boxes highlight Pk input [10% of what is used for binding] and Pk bound to “Y”). See main Figure 4B-C for competitive binding assay, demonstrating that Pk binds better to the “pY”-peptide than Dsh.
