# Supplementary material for: A Van Gogh/Vangl tyrosine phosphorylation switch regulates its interaction with core Planar Cell Polarity factors Prickle and Dishevelled
Source: PLoS Genet. 2023 Jul 18;19(7):e1010849. doi: 10.1371/journal.pgen.1010849 (PMC10381084; doi:10.1371/journal.pgen.1010849)
Supplement: S5 Fig — (DOCX) [file pgen.1010849.s005.docx]

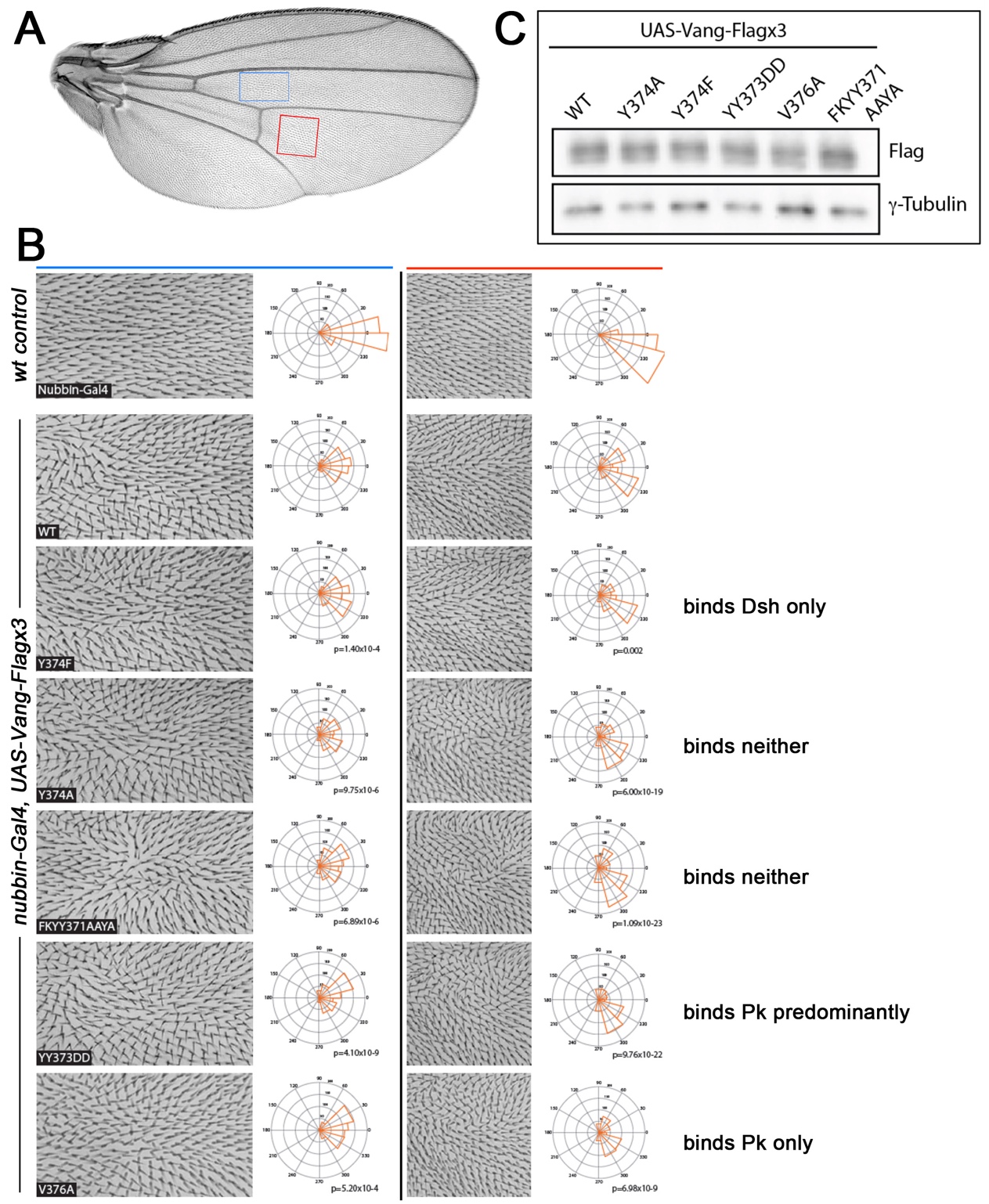


**S5 Figure (Supplement to Figure 5):**

**Distinct gain-of-function *in vivo* behavior of Vang and the respective Pk and Dsh binding mutants.**

(**A-C**) Different behavior of the individual Vang binding mutants as assayed in a GOF scenario *in vivo* in adult wings. (**A**) Wild-type wing overview picture with boxed areas of regions of interest (ROI) in blue box and red box, as shown in individual panels in (**B**) at higher magnification. (**B**) In all panels, each ROI (blue on left and red on right) was analyzed for cellular polarity angles by actin hair orientation (genotypes are as indicated, all transgenes were expressed under UAS-control with the *nubbin-Gal4* driver). Quantification of polarity angle distribution is shown on right of representative panel for each genotype. 3-6 animals were analyzed per genotype. Note that each mutant shows a specific, but distinct distribution (as compared to Vang-*WT* and other mutants; *p* values vs Vang-*WT* are shown, as determined by Chi-squared tests). For example, note different polarity distributions in Vang-Y374F (binding Dsh only) to Vang-YY373DD (binding predominantly Pk) or Vang-Y374A (binding neither Pk nor Dsh). Note also that Vang-YY373DD and Vang-V376A, cause similar reorientations, which was consistent with the notion that both these mutant Vang isoforms can bind Pk (but not Dsh). (**C**) Western blot of wing tissue from the indicated genotypes, demonstrating comparable expression levels of all Vang isoforms (wild-type and mutant) used in this study.
