# Supplementary material for: A Van Gogh/Vangl tyrosine phosphorylation switch regulates its interaction with core Planar Cell Polarity factors Prickle and Dishevelled
Source: PLoS Genet. 2023 Jul 18;19(7):e1010849. doi: 10.1371/journal.pgen.1010849 (PMC10381084; doi:10.1371/journal.pgen.1010849)
Supplement: S6 Fig — (DOCX) [file pgen.1010849.s006.docx]

**
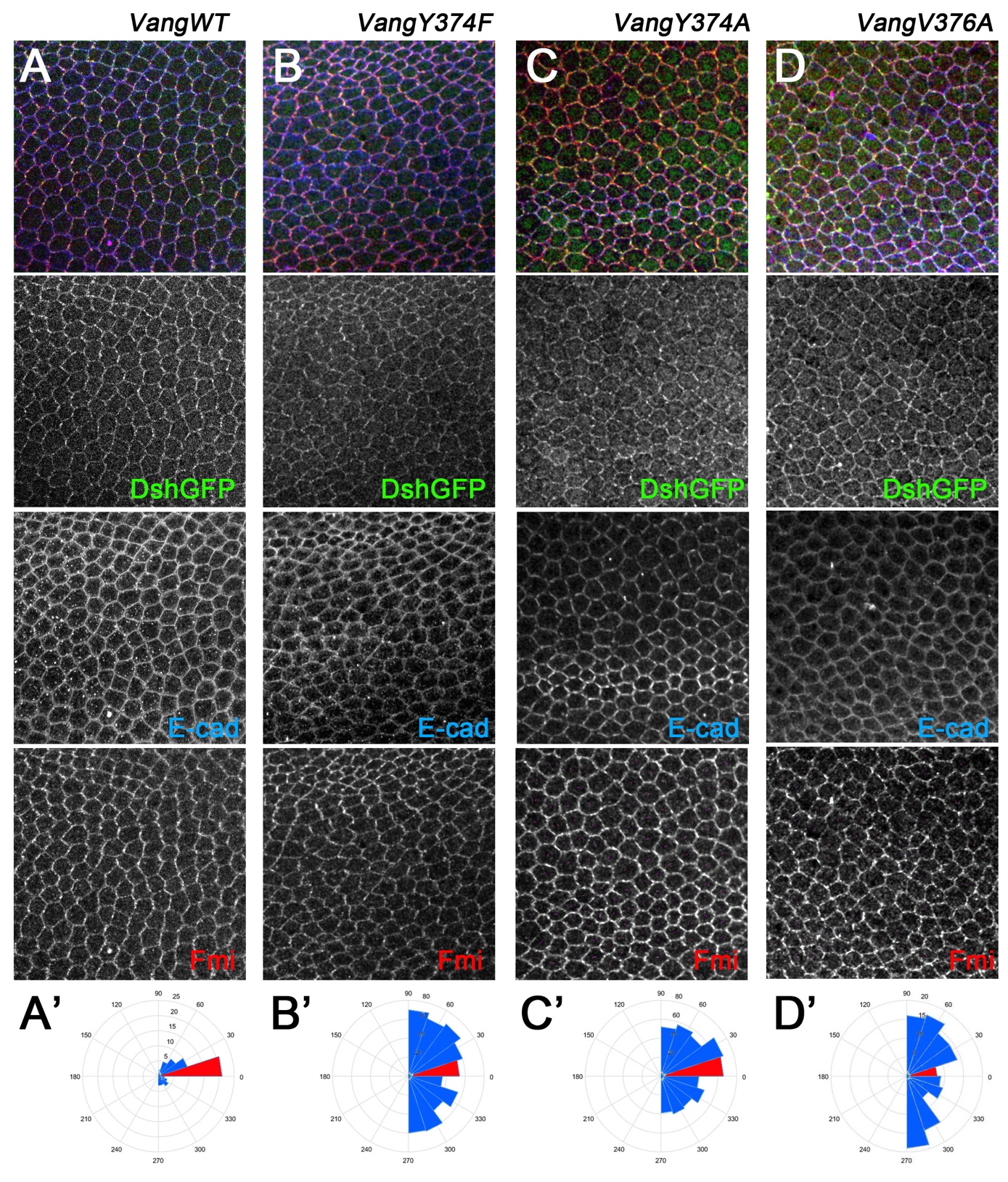
**

**S6 Figure (Supplement to Figure 6): *Vang* point mutants affect localization of core PC factors *in vivo*.**

(**A-D**) Confocal images of 28-30hr APF pupal wings of the *Vang*-/-; *tub-VangWT-Flagx3* genotype (**A**, indicated above panel as “*VangWT*”), and the three point mutants as indicated. All pupal wings are oriented horizontally with the proximal side being left and distal right, stained for DshGFP (anti-GFP, green), E-cad (blue, providing cellular outline at the junctional level), and Fmi (anti-Fmi, red). (**A**) *Vang*-/-; *tub-VangWT-Flagx3*, (**B**) *Vang*-/-; *tub-VangY374A-Flagx3*, (**C**) *Vang*-/-; *tub-VangY374F-Flagx3*, (**D**) *Vang*-/-; *tub-VangV376A-Flagx3*. Top panel shows merge of the three channels, and grayscale single channel micrographs are shown in lower panels as marked with staining. Note that Dsh is still enriched to cellular membranes in all three point mutants. Scale bar: 20μm.

(**A’-D’**) Quantification of polarity angles presented as rosette diagrams as determined by Fmi polarization in the respective genotypes indicated: (**A’**) *Vang*-/-; *tub-VangWT-Flagx3*, (**B’**) *Vang*-/-; *tub-VangY374A-Flagx3*, (**C’**) *Vang*-/-; *tub-VangY374F-Flagx3*, (**D’**) *Vang*-/-; *tub-VangV376A-Flagx3*. Note that the majority of cell orientation angles is in the proximo-distal axis (horizontal axis). Proximo-distal orientation is highlighted as red sector in the rosette diagrams in all backgrounds. While it is indistinguishable from wild-type in the *Vang*-/-; *tub-VangWT-Flagx3* background (**A’**), the cellular orientation is largely randomized in all three point mutants (**B’-D’**, similar to the *Vang* null allele).

Please note that the overall randomized polarization appearance in the mutant backgrounds is much weaker in polarization strength in general (much weaker than normal polarization in wild-type). In such a scenario the cells adopt incorrect orientation leading to waves and whorls within the polarization field, which is nonetheless somehow coordinated from cell-to-cell (through unknown mechanisms), and which is reflected in the adult wings in cellular hair orientation as for example seen in Figures 5 and S5.
