# Supplementary material for: A Van Gogh/Vangl tyrosine phosphorylation switch regulates its interaction with core Planar Cell Polarity factors Prickle and Dishevelled
Source: PLoS Genet. 2023 Jul 18;19(7):e1010849. doi: 10.1371/journal.pgen.1010849 (PMC10381084; doi:10.1371/journal.pgen.1010849)
Supplement: S7 Fig — (DOCX) [file pgen.1010849.s007.docx]

**
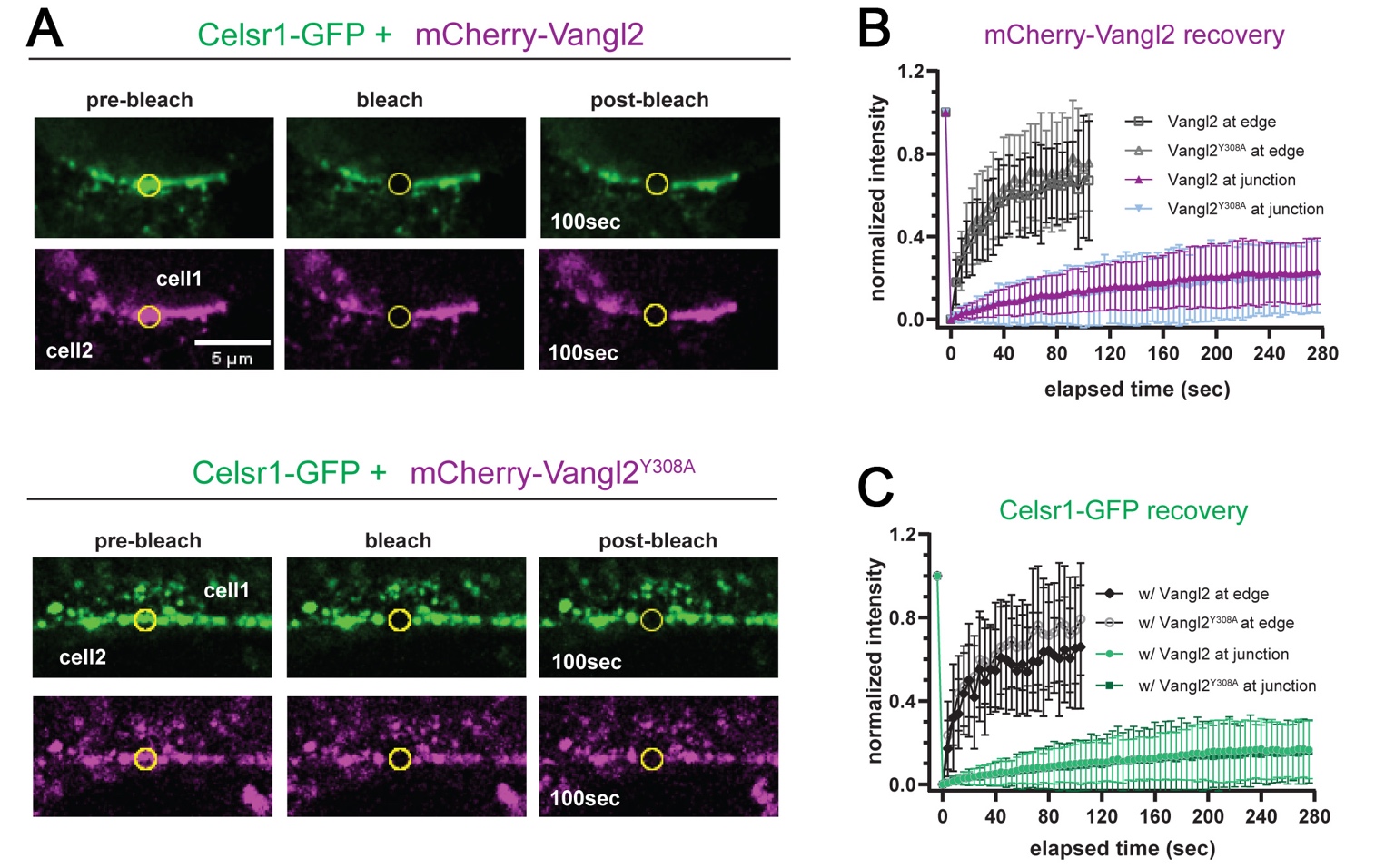
**

**S7 Figure (Supplement to Figure 6). FRAP analysis of junctional Celsr1 and Vangl2 in cultured keratinocytes.** Primary keratinocytes were co-transfected with Celsr1-GFP and mCherry-Vangl2 or mCherry-Vangl2^Y308A^. FRAP was performed on junctional regions as well as free edges of the cell.

(**A**) Representative example of junctional region between two keratinocytes expressing Celsr1-GFP (green) and mCherry-Vangl2 (magenta; top rows) or Celsr1-GFP and mCherry-Vangl2^Y308A^ (bottom rows) at pre-bleach, post-bleach and 100 seconds post-recovery timepoints (bleached ROIs depicted by yellow circle). (**B**) FRAP recovery profiles for wild type mCherry-Vangl2 (gray squares at cell edges, n=19, and magenta triangles at cell junctions, n=22) and mCherryVangl2^Y308A^ (gray triangles at cell edges, n=21, and blue inverted triangles at cell junctions, n=29). Normalized mean intensity with standard deviations are plotted versus time. (**C**) FRAP recovery profiles for Celsr1-GFP when co-expressed with wild type mCherry-Vangl2 (black diamonds at cell edges, n=17, and light green circles at cell junctions, n=20) or with mCherry-Vangl2Y308A (gray circles at cell edges, n=21, and dark green squares at cell junction, n=29). Normalized mean intensity with standard deviations of the bleach and recovery profiles are plotted versus time. Data pooled from four independent experiments.
